# Trx4, a novel thioredoxin protein, is important for *Toxoplasma gondii* fitness

**DOI:** 10.1186/s13071-024-06259-9

**Published:** 2024-04-04

**Authors:** Zhi-Wei Zhang, Meng Wang, Li-Xiu Sun, Hany M. Elsheikha, Cheng-Lin Lei, Jin-Lei Wang, Bao-Quan Fu, Jian-Xun Luo, Xing-Quan Zhu, Ting-Ting Li

**Affiliations:** 1 State Key Laboratory for Animal Disease Control and Prevention, Key Laboratory of Veterinary Parasitology of Gansu Province, Lanzhou Veterinary Research Institute, Chinese Academy of Agricultural Sciences, Lanzhou, Gansu Province 730046 People’s Republic of China; 2 Faculty of Medicine and Health Sciences, School of Veterinary Medicine and Science, University of Nottingham, Sutton Bonington Campus, Loughborough, LE12 5RD UK; 3 Institute of Urban Agriculture, Chinese Academy of Agricultural Sciences, Chengdu, Sichuan Province 610213 People’s Republic of China; 4 Laboratory of Parasitic Diseases, College of Veterinary Medicine, Shanxi Agricultural University, Taigu, Shanxi Province 030801 People’s Republic of China

**Keywords:** *Toxoplasma gondii*, Thioredoxin, Parasitophorous vacuole, Virulence, TurboID

## Abstract

**Background:**

To successfully replicate within the host cell, *Toxoplasma gondii* employs several mechanisms to overcome the host cell defenses and mitigate the harmful effects of the free radicals resulting from its own metabolic processes using effectors such as thioredoxin proteins. In this study, we characterize the location and functions of a newly identified thioredoxin in *T. gondii*, which was named Trx4.

**Methods:**

We characterized the functional role of Trx4 in *T. gondii* Type I RH and Type II Pru strains by gene knockout and studied its subcellular localization by endogenous protein HA tagging using CRISPR-Cas9 gene editing. The enzyme-catalyzed proximity labeling technique, the TurboID system, was employed to identify the proteins in proximity to Trx4.

**Results:**

Trx4 was identified as a dense granule protein of *T. gondii* predominantly expressed in the parasitophorous vacuole (PV) and was partially co-localized with GRA1 and GRA5. Functional analysis showed that deletion of *trx4* markedly influenced the parasite lytic cycle, resulting in impaired host cell invasion capacity in both RH and Pru strains. Mutation of Trx domains in Trx4 in RH strain revealed that two Trx domains were important for the parasite invasion. By utilizing the TurboID system to biotinylate proteins in proximity to Trx4, we identified a substantial number of proteins, some of which are novel, and others are previously characterized, predominantly distributed in the dense granules. In addition, we uncovered three novel proteins co-localized with Trx4. Intriguingly, deletion of *trx4* did not affect the localization of these three proteins. Finally, a virulence assay demonstrated that knockout of *trx4* resulted in a significant attenuation of virulence and a significant reduction in brain cyst loads in mice.

**Conclusions:**

Trx4 plays an important role in *T. gondii* invasion and virulence in Type I RH strain and Type II Pru strain. Combining the TurboID system with CRISPR-Cas9 technique revealed many PV-localized proximity proteins associated with Trx4. These findings suggest a versatile role of Trx4 in mediating the processes that occur in this distinctive intracellular membrane-bound vacuolar compartment.

**Graphical Abstract:**

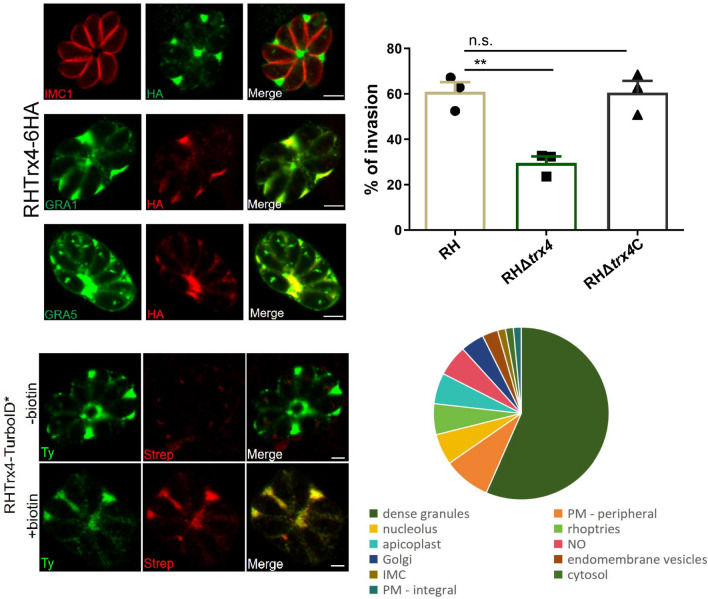

**Supplementary Information:**

The online version contains supplementary material available at 10.1186/s13071-024-06259-9.

## Background

*Toxoplasma gondii* is an obligate protozoan capable of thriving inside nearly all nucleated eukaryotic cells [1, 2]. While *T. gondii* infection is often symptomless in individuals with a competent immune system, it can pose significant health risks to the immunocompromised individuals [3, 4]. *Toxoplasma gondii* infection can also have severe adverse consequences for pregnant women and can lead to miscarriage or stillbirth [5–7].

When *T. gondii* tachyzoites invade the host cell, they establish a specialized intracellular compartment known as a parasitophorous vacuole (PV) [8]. The PV serves as a protective niche where the tachyzoites reside and replicate [9]. Within the PV, the parasites are shielded from host immune responses and can exploit the host cell’s machinery to their advantage [10, 11]. The PV not only provides a physical barrier that protects the tachyzoites from the host cell’s defenses but also plays a crucial role in nutrient acquisition to support the parasite growth and replication [12–14]. Additionally, the metabolic waste products generated by the parasites are transported out of the PV, preventing the accumulation of toxic substances that could harm the parasites [14].

A substantial body of research revealed the crucial roles of PV-localized proteins in the parasite survival, including their contribution to parasite fitness and immune modulation within the PV in the infected cell. For instance, GRA7 binds to Irga6 and promotes its multimerization, subsequently facilitating its breakdown into monomers and possibly dimers, which may undergo phosphorylation by ROP18 [15–17]. GRA7 is also an allosteric activator of ROP18 [18]. ROP5 is an important virulence factor in mice via regulating the catalytic activity of ROP18 through forming an active complex with the kinases ROP18 and ROP17 [19–21]. This complex hinders IRG recruitment to the PVs and protects the parasites from elimination in IFN-γ activated macrophages [21]. The pseudokinase ROP5 also binds to mouse Irga6 and Irgb6 and prevents their loading onto the *T. gondii*-containing PV [19, 20], Interestingly, ROP5 and ROP18 do not have a marked impact on the parasite survival in IFN-γ-activated human cells because of the lack of the immunity-related GTPase (IRG) system [20]. ROP18 phosphorylates IRGs and results in their inactivation and detachment from the PV [15, 16]. ROP39 interacts with Irgb10 and inhibits homodimer formation of GTPase, resulting in an overall decrease of IRG protein load on the parasite vacuole membrane (PVM) [22]. GRA60 contributes to the virulence of RH strain [23]. RH strain lacking GRA60 can recruit Irgb10 and Irga6 to the PVM, which is consistent with observations in RHΔ*rop5* strain [23]. MYR complexes are essential for the transport of effector GRA16 and upregulate human c-Myc and cyclin E1 in infected cells [24]. PPM3C possesses a PP2C-class serine/threonine phosphatase domain and interacts with MYR1, which contributes to the export of effector into the host cells [24, 25]. The deletion of PPM3C impairs the parasite's ability to form plaques and export GRA16 and GRA28 [25]. GRA39 was first identified in the proximity proteins of GRA17 using enzyme-catalytic proximity labeling technique (BioID system) [26]. GRA39 plays a pivotal role as a virulence factor in Type II Pru strain, and its deletion causes growth defects, lipid accumulation in the PV and the accumulation of amylopectin granules in the cytoplasm [26]. In malaria parasites, the *Plasmodium* translocon of the exported proteins (PTEX) is located in the PV and serves as the translocon for the exported proteins [27]. PTEX consists of various components, including Hsp101, PTEX150, EXP2, PTEX88 and thioredoxin (Trx) 2. PfTrx2 that was localized in the PV of malaria parasites expanded the function of Trx to include protein translocation [28].

The thioredoxin system is found in prokaryotes and eukaryotes. This system comprises thioredoxin (Trx), thioredoxin reductase (TrxR) and nicotinamide adenine dinucleotide phosphate (NADPH) [29]. Trx interacts with proteins to maintain them in a relatively stable reduced state. After reducing the target protein, Trx becomes oxidized. It is then restored to its reduced form via the action of electrons provided by TrxR and NADPH, enabling it to carry out its normal biological functions [30]. The Trx domain consists of five beta folds and four alpha helices, forming a spherical structure with a hydrophobic center. The classical active site sequence, -Cys-Gly-Pro-Cys-, is located in the Trx domain [30]. Trxs are antioxidant enzymes which play a role in the resistance to oxidative stress in organisms and contribute to other biological processes, such as protein folding, DNA synthesis, regulation of apoptosis and protein transportation [30–32].

Two Trxs, namely ATrx1 and ATrx2, have been identified in the apicoplast in *T. gondii*. Conditional knockdown of ATrx1 or ATrx2 resulted in severe defects in parasite survival, with a few plaque formations [32]. CTrp26 and CTrx1 are two other Trxs of *T. gondii*, located in the cytoplasm of the parasite; however, neither CTrp26 nor CTrx1 plays a key role in parasite growth and virulence [33].

In this study, we performed a comprehensive characterization of a previously unknown thioredoxin named Trx4 (TGME49_224060) in *T. gondii*. Trx4 was found to be localized in the PV. In addition, parasites lacking Trx4 exhibited a marked impairment in growth with a limited ability to invade host cells, and Trx domains were found to play a role in the parasite invasion. Furthermore, we employed the TurboID technique to biotinylate proteins located in close proximity to Trx4. Most of the identified proximity proteins were localized in the PV, and three novel proximity proteins were characterized. The deletion of *trx4* significantly attenuated the virulence of *T. gondii* in mice. Our data show that Trx4 plays a pivotal role in *T. gondii* growth and serves as a virulence factor.

## Methods

### Parasite and cell culture

Strains of *T. gondii* and human foreskin fibroblast (HFF) cells were maintained in Dulbecco’ modified Eagle’s medium (DMEM) supplemented with 2% fetal bovine serum (FBS), penicillin (100 U/ml) and streptomycin (100 μg/ml), under 37 ℃ and 5% CO_2_ atmosphere. RHΔ*ku80* and PruΔ*ku80* were used to construct C-terminal strains and knockout strains.

### Construction of C-terminal, knockout and complemented strains

To generate C-terminal strains, a 6 × HA tag or a TurboID-4Ty tag containing a 42-bp short homologous arm was amplified from pLIC-6 × HA-DHFR or pLIC-TurboID-4Ty-DHFR, respectively. A single guide RNA (SgRNA) was designed in proximity to the STOP codon of the *trx4* gene. The SgRNA for the C-terminal strain and the 6 × HA fragment or TurboID-4Ty tag fragment were introduced into the parental parasite via electroporation. Positive strains were selected via screening using 3 μM pyrimethamine and limiting dilution. Confirmation of successful integration was achieved using DNA sequencing, indirect immunofluorescence assays (IFA) and Western blotting.

To generate knockout strains, 5′- and 3′-homologous arms of *trx4* amplified from the genomic DNA of the wild-type (wt) parasites were cloned into the pUC19 plasmid with DHFR fragment. The 5′UTR-DHFR-3’UTR fragment and SgRNA targeting *trx4* were transfected into parental parasites by electroporation. Stable strains were selected as mentioned above.

To generate complemented strains containing Trx4 fused with a 6 × HA tag at the C-terminus, the complemented fragment was amplified from the complemented p*trx4*-Trx4-6HA-*dhfr*-ter plasmid, which contains Trx4 promoter, *trx4* cDNA fused with a 6 × HA tag, a DHFR terminator and a CAT marker. To construct a complemented strain with Trx4 but without a fusion tag, a complemented fragment was amplified from the complemented p*trx4*-Trx4-*trx4*-ter plasmid, which contains Trx4 promoter, *trx4* DNA*,* a Trx4 terminator and a CAT marker. This complemented fragment and SgRNA targeting *uprt* were transfected into RHΔ*trx4* or PruΔ*trx4* strain. To investigate the role of the Trx domain, the first and/or second Trx domains were deleted from the p*trx4*-Trx4-*trx4*-ter plasmid using the Q5 site-directed mutagenesis kit (New England Biolabs). Stable strains were selected by chloramphenicol and 5-fluorodeoxyuridine. Positive clones were confirmed by DNA sequencing, IFA and Western blotting. All primers used are listed in Additional file 1: Table S1.

### Indirect immunofluorescence analysis and Western blotting

For IFA, tachyzoites were allowed to infect HFF cells for 24 h. Subsequently, the parasites were fixed with 4% paraformaldehyde (PFA) and permeabilized using 0.1% Triton X-100 solution. Primary antibodies used were mouse anti-SAG1 (1:1000), rabbit anti-HA (1:500), mouse anti-HA (1:500), rabbit anti-IMC1 (1:500), mouse anti-IMC1 (1:500), rabbit anti-MIC2 (1:500), rabbit anti-ACP (1:500), rabbit anti-hsp60 (1:500), mouse anti-GRA1 (1:500), rabbit anti-GRA5 (1:500) and mouse anti-Ty (1:1000).

For Western blotting, the pellets of tachyzoites were treated with RIPA buffer supplemented with protease inhibitor cocktail and EDTA. The supernatant of lysate was used for SDS-PAGE analysis and transferred to PVDF member. Primary antibodies used in Western blotting were rabbit anti-HA (1:1000), mouse anti-Ty (1:1000) and rabbit anti-aldolase (1:500).

### Phenotypic analyses: plaque assay, invasion assay, replication assay and egress assay

For the plaque assay, approximately 200 tachyzoites of RH strain or Pru strain were used to infect HFF cells grown in 12-well tissue culture plastic plates. Parasites were cultured at 37 ℃ with 5% CO_2_ atmosphere for 7 days (RH strain) or 10 days (Pru strain). Then, the samples were fixed with 4% PFA and stained with 0.5% crystal violet for 15 min. The plaque sizes were measured and analyzed by Image J software. The experiment was performed three independent times.

For the parasite invasion assay, approximately 5 × 10^5^ tachyzoites of wt, knockout or complemented strains were used to infect HFFs for 40 min (RH strain) or 1 h (Pru strain). After invasion, parasites were fixed with 4% PFA. Extracellular parasites were stained with mouse anti-SAG1 and Alexa Fluor 594 goat anti-mouse IgG. Following permeabilization with 0.1% Triton X-100, all tachyzoites, including extracellular and intracellular parasites, were stained with rabbit anti-IMC1 and Alexa Fluor 488 goat anti-rabbit IgG. Invasion efficiency was determined by divining the number of intracellular parasites by the total number of parasites. We randomly selected five microscopic fields at the four corners and the center of the respective wells of each sample and counted at least 100 tachyzoites. Invasion assays were performed in biological triplicate.

For the replication assay, approximately 3 × 10^5^ tachyzoites were inoculated into HFF cells for 4 h (RH strain) or 5 h (Pru strain). Then, the samples were washed by DMEM twice to remove extracellular parasites and were cultured for further 20 h (RH strain) or 27 h (Pru strain). Parasites were fixed as above and counted under an inverted microscope. At least 150 PVs from three biological replicates were examined to determine the number of tachyzoites per PV.

For the egress assay, approximately 2 × 10^5^ tachyzoites were used to infect HFF cells for 4 h (RH strain) or 5 h (Pru strain) and were washed by DMEM to remove tachyzoites that remained extracellular. When most of the PVs contained 32 tachyzoites, infected cells were treated with 3 μM A23187 for 3 min to induce parasite egress. Samples were fixed and counted under an inverted microscope. Egress efficiency was determined by dividing the number of lysed PVs by all PVs, and at least 100 PVs were counted. The experiments were performed independently three times.

### Enrichment of biotinylated proteins

BirA*, a biotin protein ligase with a molecular size of 35 kDa, serves as a core component of the BioID (proximity-dependent biotin identification) system. This system catalyzes the biotinylation of proteins in close proximity to the target protein [34]. The BioID system utilizes non-toxic biotin as its sole substrate, rendering it suitable for a wide range of in vitro applications [35]. However, the slow kinetics of BirA poses a limitation, requiring 18–24 h for full protein biotinylation, which is not ideal for transient screening [36]. To address this limitation, the TurboID system was developed as an advanced version of BioID, enabling high-efficiency labeling within a shorter biotinylation time (< 10 min) [34]. TurboID is particularly advantageous for identifying proximity proteins in the mitochondrial matrix and endoplasmic reticulum lumen [37].

In this study, we employed the TurboID system to investigate the proximity proteins of Trx4. Endogenous Trx4 was C-terminally fused with a TurboID-4Ty tag. Upon addition of biotin, a substrate of the TurboID enzyme, the proximity proteins of Trx4 were labeled with biotin. These biotin-labeled proteins can be detected using antibodies such as streptavidin-horseradish peroxidase conjugate (Strep-HRP) and can be captured by streptavidin beads for subsequent mass spectrometry analysis.

For IFA, the RHTrx4-TurboID* strain was incubated in a cell dish for 22 h. Subsequently, the culture medium was replaced with 2% DMEM containing 200 μM D-biotin, and the incubation continued for an additional 2 h. Hereafter, the samples were prepared for IFA. In the IFA procedure, antibodies employed included mouse anti-Ty (1:1000) and streptavidin-Alexa Fluor 594 conjugate (Strep-594).

For Western blotting analysis of the biotinylated proteins, RH strain or RHTrx4-TurboID* strain was cultured in a 25T flask with or without the addition of 200 μM D-biotin. The four kinds of freshly egressed parasites (RH strain incubated with or without D-biotin and RHTrx4-TurboID* strain incubated with or without D-biotin) were collected by centrifugation and washed with PBS. The pellet containing the tachyzoites was treated with RIPA buffer supplemented with a protease inhibitor cocktail and subsequently subjected to SDS-PAGE analysis. The detection of the bands was achieved by using mouse anti-Ty (1:1000) antibodies and Strep-HRP.

For mass spectrometry analysis of the biotinylated proteins, the RHTrx4-TurboID* or RH parasites were cultured in a 175 T flask containing 200 μM D-biotin for approximately 40 h. RH parasites were used as negative control. Prior to egressing from host cells, the parasites were harvested and washed with chilled PBS. The resulting pellet of tachyzoites was subjected to treatment with RIPA buffer supplemented with a protease inhibitor cocktail for 40 min on ice. The lysate was incubated with streptavidin beads at 4 ℃ overnight. The streptavidin beads were then washed three times with RIPA buffer and once with chilled PBS. Finally, they were resuspended in PBS and stored at − 80 ℃ for LC–MS analysis as previously described [38].

### Bradyzoite differentiation in vitro

Approximately 2 × 10^5^ tachyzoites of Pru or PruΔ*trx4* strain were inoculated into cell culture dishes containing monolayer of HFF cells and incubated at 37 ℃ with 5% CO_2_ atmosphere for 5 h. Then, samples were washed by DMEM twice to remove non-attached parasites and were divided into two groups. One group was cultured with differentiation medium (pH = 8.2, ambient CO_2_) for 2 days; the other group was cultured at 5% CO_2_ and 37 ℃ for 2 days. Subsequently, the samples were used to perform IFA, and all parasites were stained with rabbit anti-IMC1 primary antibody and goat anti-rabbit Alexa Fluor^®^ 594 secondary antibody, and the tissue cysts were stained with FITC-conjugated *Dolichos biflorus* agglutinin (DBA). The samples were imaged and counted using Leica confocal microscope [39]. The experiment was repeated three independent times.

### Virulence assay

Six- to 7-week-old female SPF Kunming mice were purchased from the Center of Laboratory Animals, Lanzhou Veterinary Research Institute. Mice were housed under a 12-h light/12-h dark cycle in separate cages and fed with fresh water and a standard chow diet. The mice were euthanized when they reached their humane endpoint. For the acute infection assay, mice were intraperitoneally (i.p.) infected by 200 tachyzoites of RH, RHΔ*trx4* or RHΔ*trx4*C. The survival rate and clinical signs were recorded for 30 days. For the chronic infection assay, mice were infected i.p. by a low dose (2 × 10^2^ tachyzoites) or high dose (2 × 10^4^ tachyzoites) of the Pru strain. The surviving mice were confirmed to be infected by serological testing. To assess the cyst-forming ability of PruΔ*trx4*, mice that survived were euthanized at 30 days post infection, and the brain cyst burdens were measured.

### Statistical analysis

GraphPad Prism 5.0 (GraphPad Software) was used for statistical analysis and to generate the graphs. Student’s *t* test was used to evaluate the intergroup differences. Survival curves of mice were analyzed by Gehan-Breslow-Wilcoxon test. The differences were considered statistically significant when the *p* value was < 0.05. The experiments were performed at least three independent times.

## Results

### Trx4 was localized in the PV of *T. gondii*

**Fig. 1:**
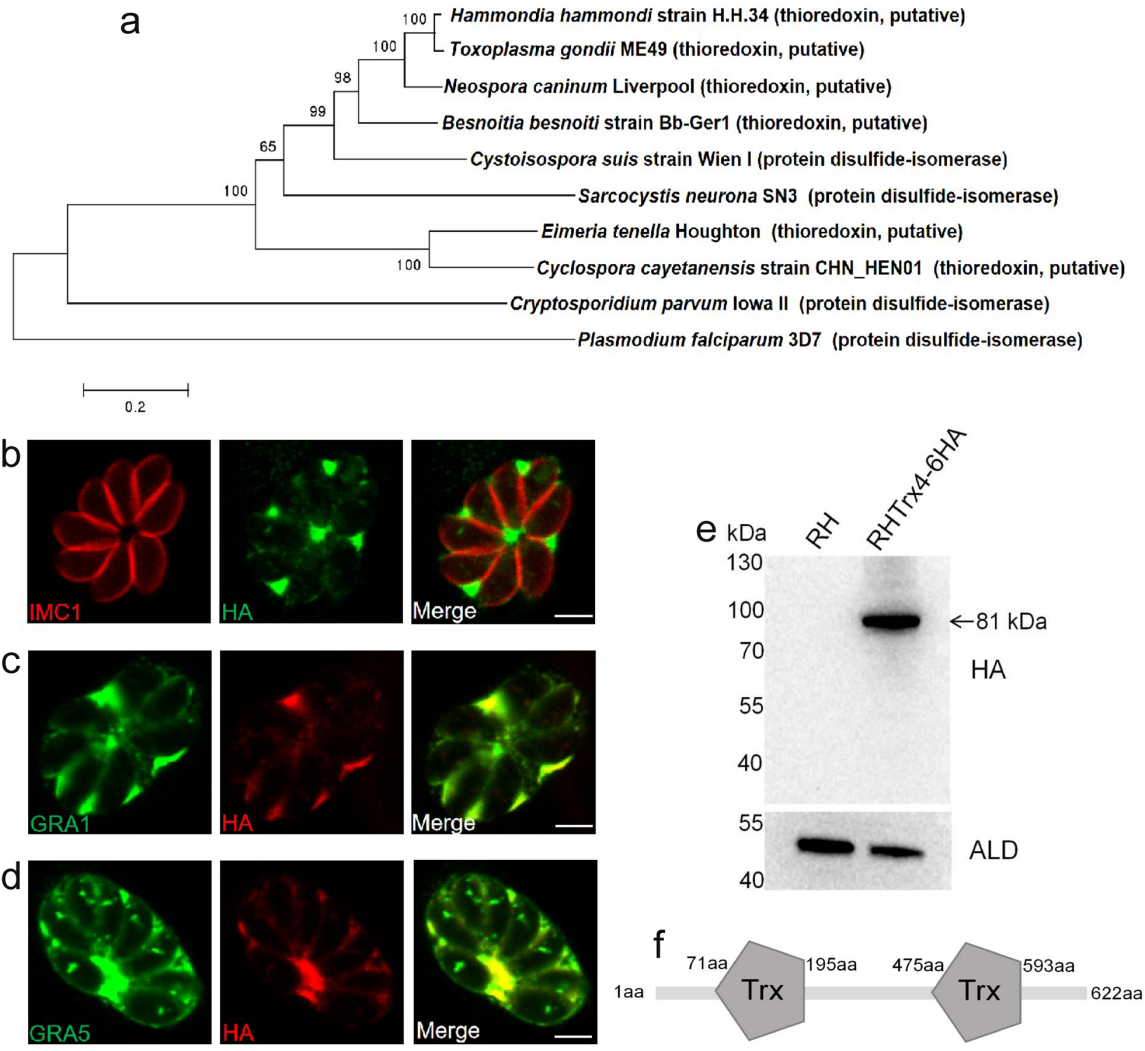
The subcellular localization of Trx4 in *Toxoplasma gondii*. **a** Phylogenetic analysis of Trx4 proteins in *T. gondii* (TGME49_224060), *Hammondia hammondi* (HHA_224060), *Neospora caninum* (NCLIV_048460), *Besnoitia besnoiti* (BESB_079560), *Cystoisospora suis* (CSUI_002634), *Eimeria tenella* (EPH_0016720), *Cyclospora cayetanensis* (cyc_05007), *Sarcocystis neurona* (SN3_01700400), *Cryptosporidium parvum* (cgd1_800) and *Plasmodium falciparum* (PF3D7_1472600). The phylogenetic tree was constructed by the neighbor-joining method. **b** IFA revealed that Trx4 was localized in the PV. **c** and **d** Trx4 co-localized with GRA1 (**c**) or GRA5 (**d**). Green, rabbit anti-HA, mouse anti-GRA1 or rabbit anti-GRA5; red, mouse anti-IMC1, rabbit anti-HA or mouse anti-HA. Scale bar: 3 μm. **e** Western blotting confirmed that Trx4-6HA strain was successfully generated, and a single protein band was visualized. HA, rabbit anti-HA; ALD, rabbit anti-aldolase. **f** Trx4 was predicted to have two Trx domains, located at amino acid positions 71–195 and 475–593, respectively, as indicated by (https://prosite.expasy.org/)

At least 15 Trxs or Trx-domain containing proteins were identified in the ToxoDB (https://toxodb.org) (Additional file 2: Table S2), which were predicated to have different locations in *T. gondii*. In this study, the novel thioredoxin (TGME49_224060), named Trx4, was selected and studied. Phylogenetic analysis revealed that this Trx was widely present in the Apicomplexan parasites, including *T. gondii* (TGME49_224060)*, Hammondia hammondi* (HHA_224060), *Neospora caninum* (NCLIV_048460), *Besnoitia besnoiti* (BESB_079560), *Cystoisospora suis* (CSUI_002634), *Eimeria tenella* (EPH_0016720), *Cyclospora cayetanensis* (cyc_05007), *Sarcocystis neurona* (SN3_01700400), *Cryptosporidium parvum* (cgd1_800) and *Plasmodium falciparum* (PF3D7_1472600) and TgTrx4 was more closely related to *H. hammondi* Trx (Fig. 1a). To characterize the subcellular localization of TgTrx4, a 6 × HA tag was introduced at the C-terminus of Trx4 using the CRISPR-Cas9 system (Additional file 3: Fig. S1). This modification was validated by using IFA and Western blotting (Fig. 1). The results of the IFA demonstrated that Trx4 was primarily localized in the PV (Fig. 1b). To confirm this finding, classical dense granule proteins, GRA1 and GRA5, were stained using anti-GRA1 and anti-GRA5 antibodies in the Trx4-6HA strain. There was evident partial co-localization between Trx4 and GRAs (GRA1 and GRA5), providing additional evidence supporting that Trx4 was a dense granule protein (Fig. 1c and d). RHTrx4-6HA strain was also stained with rabbit anti-ACP, rabbit anti-hsp60 or rabbit anti-MIC2 to explore the possibility of localization in other organelles, such as TgATrx1 and TgATrx2, which are localized in the apicoplast [32]. The results of IFA revealed that Trx4 was not localized at the apicoplast, mitochondrion or microneme (Additional file 3: Fig. S1). Western blotting corroborated the successful expression of Trx4 fused with a 6 × HA tag, with a single protein band detected at the expected molecular weight of ~ 81 kDa (Fig. 1e). Trx4 was composed of 622 amino acids and was predicted to contain two Trx domains (https://prosite.expasy.org/), which were located at amino acid positions 71–195 and 475–593, respectively (Fig. 1f).

### Deletion of *trx4* reduced the parasite invasiveness in both RH and Pru strains

**Fig. 2:**
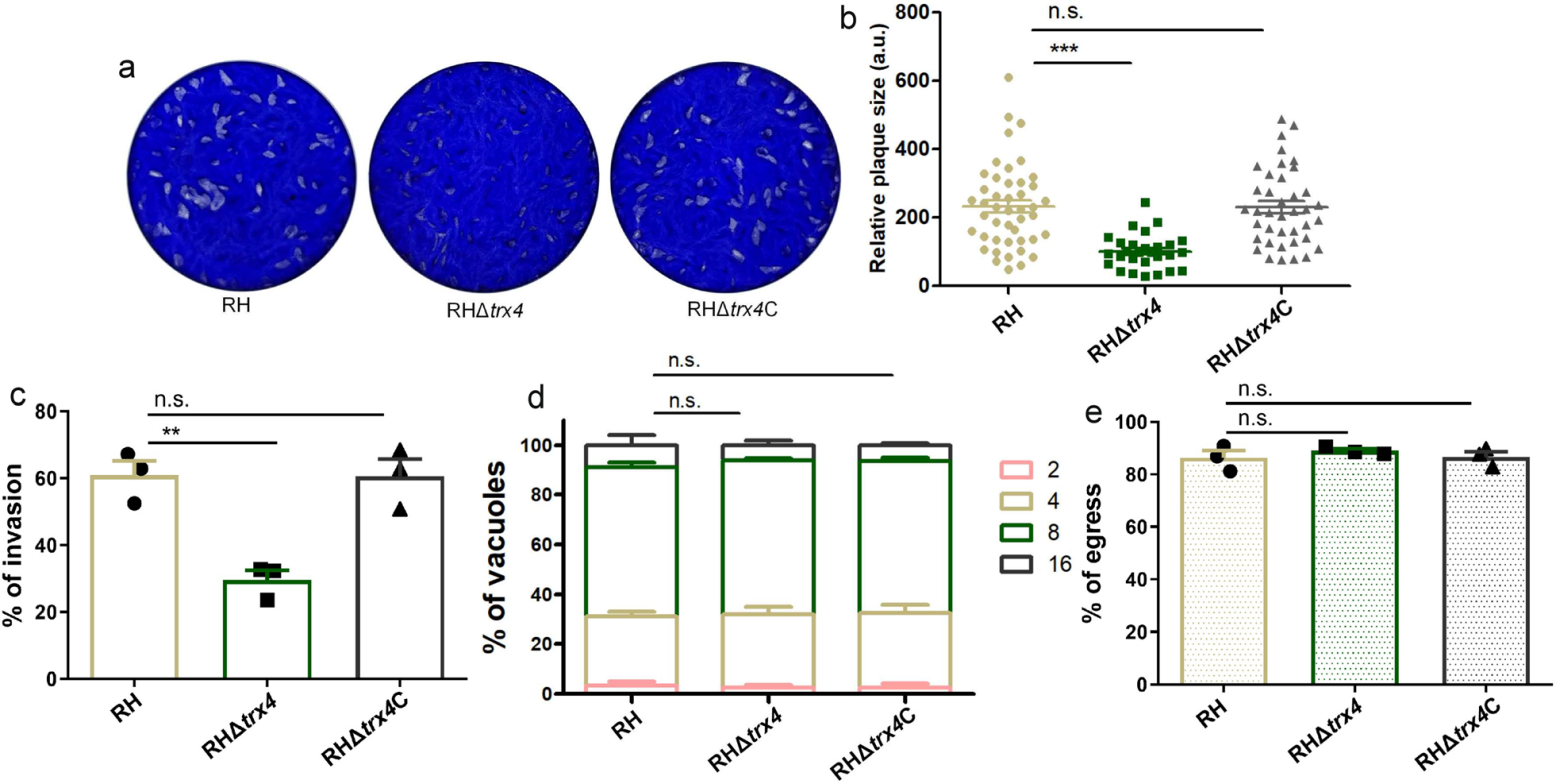
Phenotypic analysis of RHΔ*trx4*. **a** The plaque assays of RH, RHΔ*trx4* and RHΔ*trx4*C strains. **b** The relative plaque sizes of RH, RHΔ*trx4* and RHΔ*trx4*C strains measured from (**a**). The experiments were performed three independent times. The difference was analyzed by Student’s *t*-test. ***P < 0.001; n.s., not significant. **c** Differences in the host cell invasion in RH, RHΔ*trx4* and RHΔ*trx4*C strains. Five microscopic fields were randomly selected in each sample. The experiments were performed three independent times. The difference was analyzed by Student’s *t*-test. **P < 0.01; n.s., not significant. **d** Differences in the intracellular replication in RH, RHΔ*trx4* and RHΔ*trx4*C strains grown in HFFs for 24 h. The experiments were performed three independent times. The difference was analyzed by Student’s *t*-test. n.s., not significant. **e** Differences in the egress between RH, RHΔ*trx4* and RHΔ*trx4*C strains. The experiments were performed three independent times. The difference was analyzed by Student’s *t*-test. n.s., not significant

To investigate the fundamental role of Trx4 in *T. gondii*, *trx4* gene was successfully disrupted by CRISPR-Cas9 system to generate RHΔ*trx4* strain, which was confirmed by diagnostic PCR (Additional file 4: Fig. S2). Plaque assay was used to examine the effect of deletion of *trx4* gene on the lytic cycle of parasites. The results showed that the size of plaques that was generated by knockout (KO) strain was smaller than that generated by wt strain, suggesting that Trx4 is important for the lytic cycle of *T. gondii* Type I strain (Fig. 2a and b). To ascertain which steps in the infection process were perturbed by the loss of *trx4*, we assessed the parasite invasion, intracellular replication and egress. The cell invasion ability of wt and KO parasites exhibited a significant difference, indicating that the deletion of *trx4* impaired the ability of tachyzoites of RH strain to invade the host cells (Fig. 2c). However, the replication of wt strain was similar to that of the KO strain, suggesting that Trx4 does not play a significant role in the endodyogeny process of *T. gondii* in RH strain (Fig. 2d). To induce parasite egress, calcium ionophore was used, and the results demonstrated that the majority of tachyzoites from RH and KO strains egressed from the PVs within 3 min (Fig. 2e).

We attempted to generate complemented strains to confirm the phenotype observed in RHΔ*trx4* strain. Unfortunately, the complemented strain RHΔ*trx4*C-6HA with Trx4 fused with a 6 × HA tag at the C-terminus and a DHFR terminator only restored the expression of Trx4 (Additional file 4: Fig. S2) but did not restore its function (Additional file 4: Fig. S2). This lack of functional restoration may be attributed to potential alterations in the function of Trx4 caused by the presence of the HA tag or the heterologous terminator. To amend this defect, we generated a new complemented strain RHΔ*trx4*C without 6 × HA Tag, and the self-terminator was used; interestingly, RHΔ*trx4*C exhibited a restored function (Fig. 2a–c), suggesting that the expression of Trx4 fused with a 6 × HA tag or the heterologous terminator affects its function. Alternatively, we generated new *trx4* knockout strains using a different guide RNA (gRNA) and a distinct homology repair template, which was confirmed by diagnostic PCR (Additional file 4: Fig. S2). This newly generated strain exhibited plaque formation and invasion defects comparable those observed in the original line (Additional file 4: Fig. S2).

Furthermore, a Type II PruΔ*trx4* strain was generated using the CRISPR-Cas9 system. Phenotypic analyses revealed that deletion of *trx4* impaired the ability of the Pru strain to generate plaques and to invade the host cells (Additional file 5: Fig. S3). On the other hand, *trx4* disruption did not affect the ability of the Pru strain to replicate and egress from the host cells (Additional file 5: Fig. S3). The PruΔ*trx4*C-6HA strain only succeeded in restoring the expression of Trx4 but did not restore its function, whereas PruΔ*trx4*C successfully restored the defect (Additional file 5: Fig. S3). These findings suggest that the observed growth defects were primarily attributed to the loss of Trx4 and were not the result of off-target effects.

### Trx domain was important for parasite invasion

**Fig. 3:**
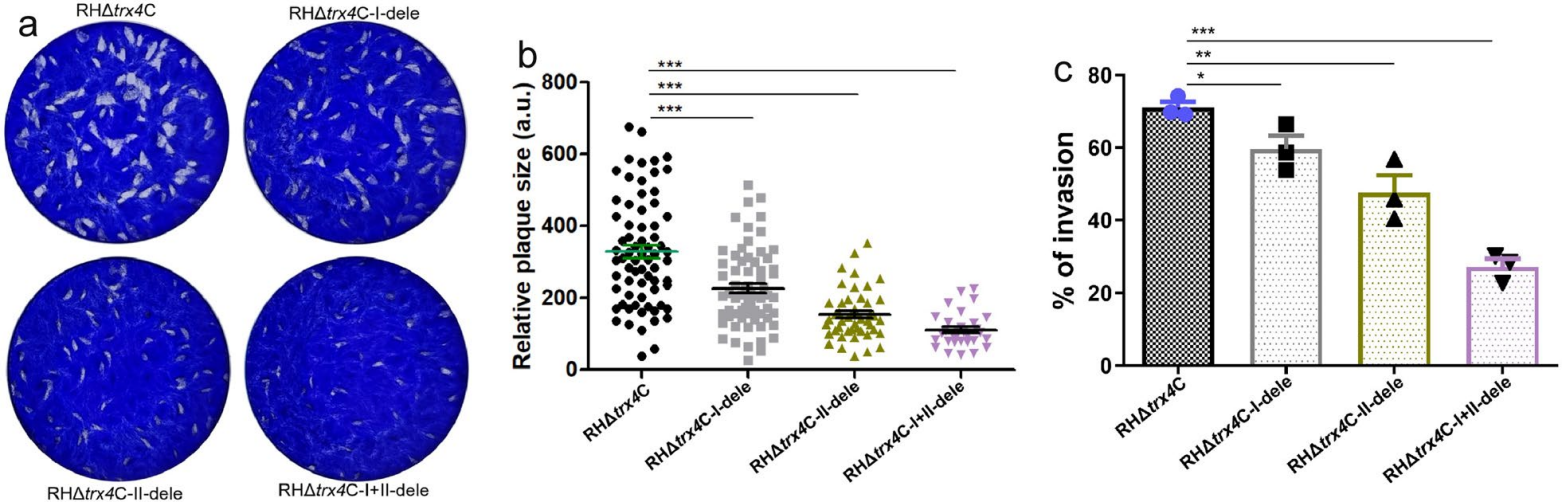
Phenotypic analyses of mutation of Trx domains in Trx4 in RH strain. **a** The plaque assays of RHΔ*trx4*C, RHΔ*trx4*C-I-dele, RHΔ*trx4*C-II-dele and RHΔ*trx4*C-I + II-dele strains. **b** The relative plaque sizes of RHΔ*trx4*C, RHΔ*trx4*C-I-dele, RHΔ*trx4*C-II-dele and RHΔ*trx4*C-I + II-dele that were measured from (**a**). The experiments were performed three independent times. The difference was analyzed by Student’s *t*-test. ***P < 0.001. **c** Differences in the host cell invasion between RHΔ*trx4*C, RHΔ*trx4*C-I-dele, RHΔ*trx4*C-II-dele and RHΔ*trx4*C-I + II-dele strains. The experiments were performed three independent times. The difference was analyzed by Student’s *t*-test. *P < 0.05; **P < 0.01; ***P < 0.001

Since Trx4 was predicted to contain two Trx domains, we explored whether these domains affect the functions of Trx4. Therefore, three Trx-domain mutants were generated, namely RHΔ*trx4*C-I-dele, RHΔ*trx4*C-II-dele or RHΔ*trx4*C-I + II-dele, which involved deleting the first Trx domain, second Trx domain, or both Trx domains, respectively (Additional file 6: Fig. S4). Plaque assays showed that both single and double deletions of Trx domains affected the ability of the complemented strains to form plaques (Fig. 3a and b). Invasion assays revealed that the deletion of the first domain, second domain or both domains significantly reduced the invasion ability of RHΔ*trx4*C (Fig. 3c), suggesting that the Trx domain is important for the biological function of Trx4.

### Biotinylation of Trx4-TurboID^*^ revealed several PV-localized proteins

**Fig. 4:**
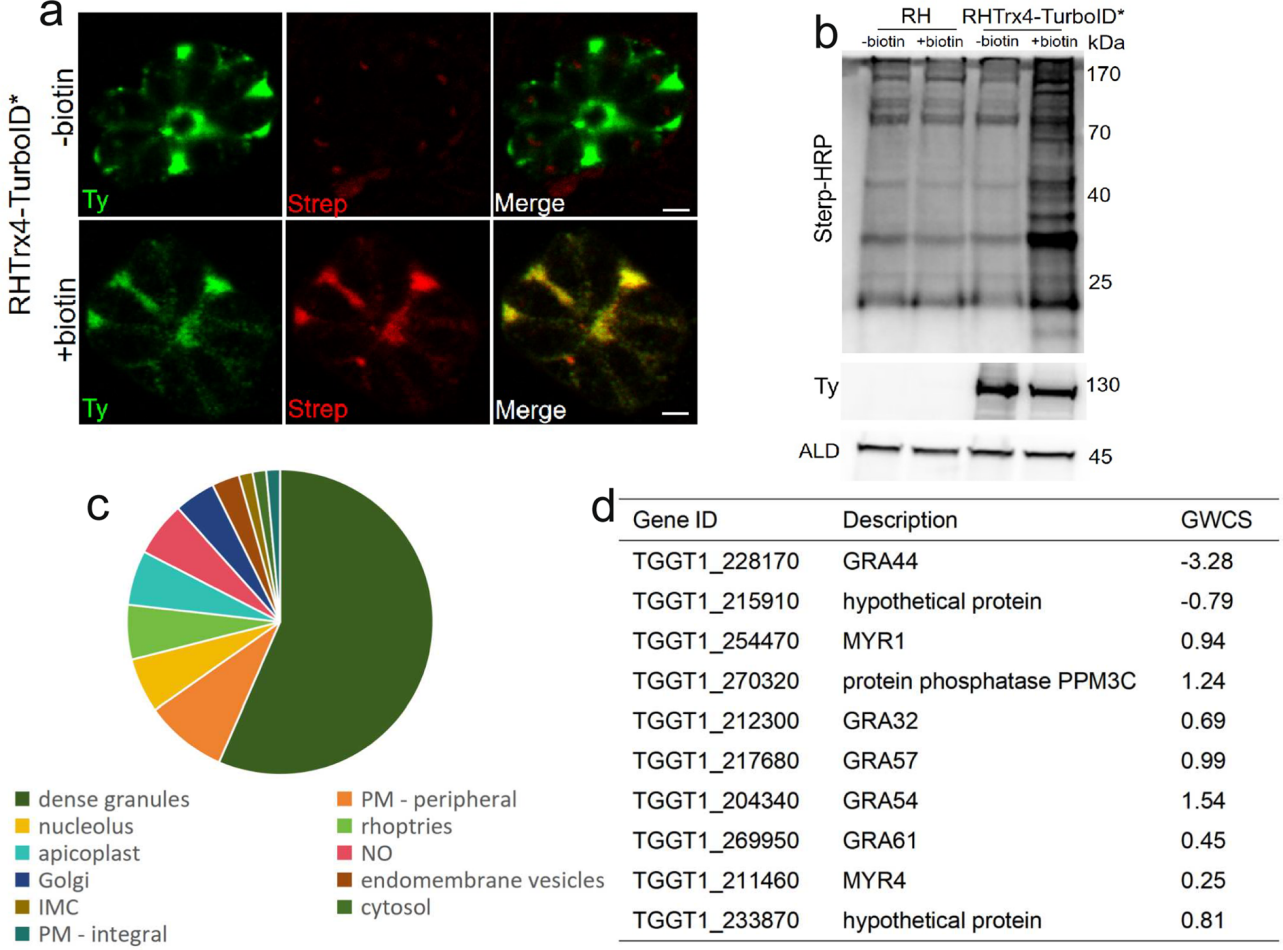
Biotinylation of the proximity proteins associated with Trx4. **a** IFA showed that Trx4 fused with the TurboID-4Ty tag was localized in the PV and proximity proteins associated with Trx4 were labeled by Strep-594 when parasites were biotinylated with D-biotin. Green, mouse anti-Ty; red, streptavidin-Alexa Fluor 594 conjugate. Scale bar: 2 μm. **b** Western blotting confirmed that biotinylation of proximity proteins were stained with Strep-HRP when parasites were incubated with D-biotin. − biotin, parasites were not incubated by D-biotin; + biotin, parasites were incubated by D-biotin; Strep-HRP, streptavidin-horseradish peroxidase conjugate; Ty, mouse anti-Ty; ALD, rabbit anti-aldolase. **c** The pie chart depicts the percentage of biotinylated proteins identified as proximity proteins of Trx4, categorized based on their predicted localization from ToxoDB. (**d**) The table summarizes the top 10 hits from mass spectrometry based on the number of unique peptides identified. GWCS, phenotype score determined based on a genome-wide CRISPR/Cas9 screening

Considering the PV localization of Trx4, we introduced a TurboID tag containing 4 × Ty at the C-terminus of Trx4 to identify the proximity proteins. Trx4, when expressed with the TurboID tag, exhibited a distribution pattern in the PV consistent with that of the Trx4-6HA strain (Figs. 1a and 4a). Subsequently, following incubation with 200 μM D-biotin, the candidate proximity proteins of Trx4 were biotinylated and stained using Strep-594, and their distribution also overlapped with that of Trx4 (Fig. 4a). Western blotting further confirmed the successful construction of Trx4-TurboID* with biotinylated proteins being labeled by Strep-HRP when Trx4-TurboID* strain was incubated by D-biotin (Fig. 4b). Meanwhile, Trx4-TurboID* strain without D-biotin treatment or RH strain incubated with D-biotin or not showed less protein that was labeled by Strep-HRP (Fig. 4b). The enrichment of biotinylated proteins was assessed using mass spectrometry, and the parental line incubated by D-biotin was used as a negative control. Combining the results of negative controls and unique peptide numbers in the treatment groups, 69 proteins were identified as being proximal to Trx4. These proteins were selected based on the presence of at least two unique peptides in every independent replicate of the treatment group and nearly 0 unique peptides in every independent replicate of the control group, as previously described [40]. As expected, the majority (39/69) of the proximity proteins were located at the dense granules including GRA9, GRA12B, GRA15, GRA16, GRA17, GRA18, GRA22, GRA32, GRA35, GRA38, GRA39, GRA44, GRA52, GRA54, GRA56, GRA57, GRA59, GRA61, GRA63, GRA70, MYR1, MYR3, MYR4 and others. In addition, several proteins distributed at other organelles were also identified, including nucleolus, rhoptries and others (Fig. 4c and d) (Additional file 7: Table S3).

### Verification of three biotinylated proteins that were co-localized with Trx4

**Fig. 5:**
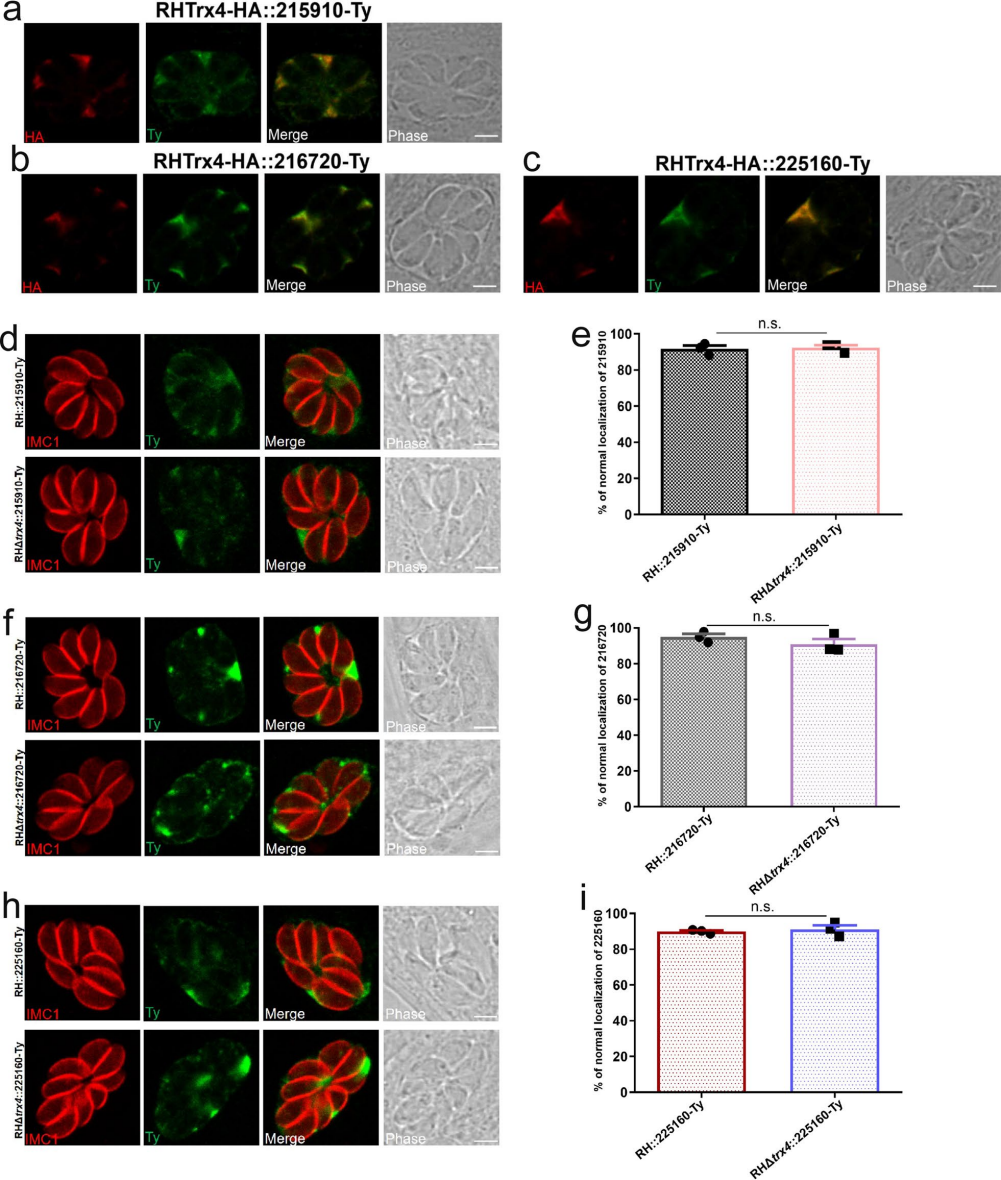
Identification of three novel proteins from the data sets of Trx4-TurboID* strain. **a**–**c** IFA showed that TGGT1_215910 (**a**), TGGT1_216720 (**b**) or TGGT1_225160 (**c**) was co-localized with Trx4. Red, rabbit anti-HA (Trx4); green, mouse anti-Ty (proximity proteins). Scale bar: 3 μm. **d**–**i** IFA and quantification of the indicated parasite strains revealed that deletion of *trx4* did not change the localization of TGGT1_215910 (**d** and **e**), TGGT1_216720 (**f** and **g**) and TGGT1_225160 (**h** and **i**). Red, mouse anti-IMC1; green, mouse anti-Ty. Scale bar: 3 μm. The experiments were performed three independent times. The difference was analyzed by Student’s *t*-test. n.s., not significant

To validate the results obtained by mass spectrometry, we selected three hypothetical proteins (TGGT1_215910, TGGT1_216720 and TGGT1_225160) that had not been previously studied. We determined the localization of these proximity proteins by employing the CRISPR-Cas9 system to insert a 2 × Ty tag at the C-terminus of their respective genes in the Trx4-6HA background. IFA revealed that these hypothetical proteins were indeed distributed in the PV and exhibited co-localization with Trx4 (Fig. 5a–c). To further explore the relationship between Trx4 and these three selected proteins, we endogenously tagged all three genes with Ty tag in the RH background or RHΔ*trx4* background. The results of IFA and statistical analysis indicated that deletion of *trx4* did not influence the localization of TGGT1_215910 (Fig. 5d and e), TGGT1_216720 (Fig. 5f and g) or TGGT1_225160 (Fig. 5h and i).

### Knockout of* trx4* did not alter the location of dense granule proteins

**Fig. 6:**
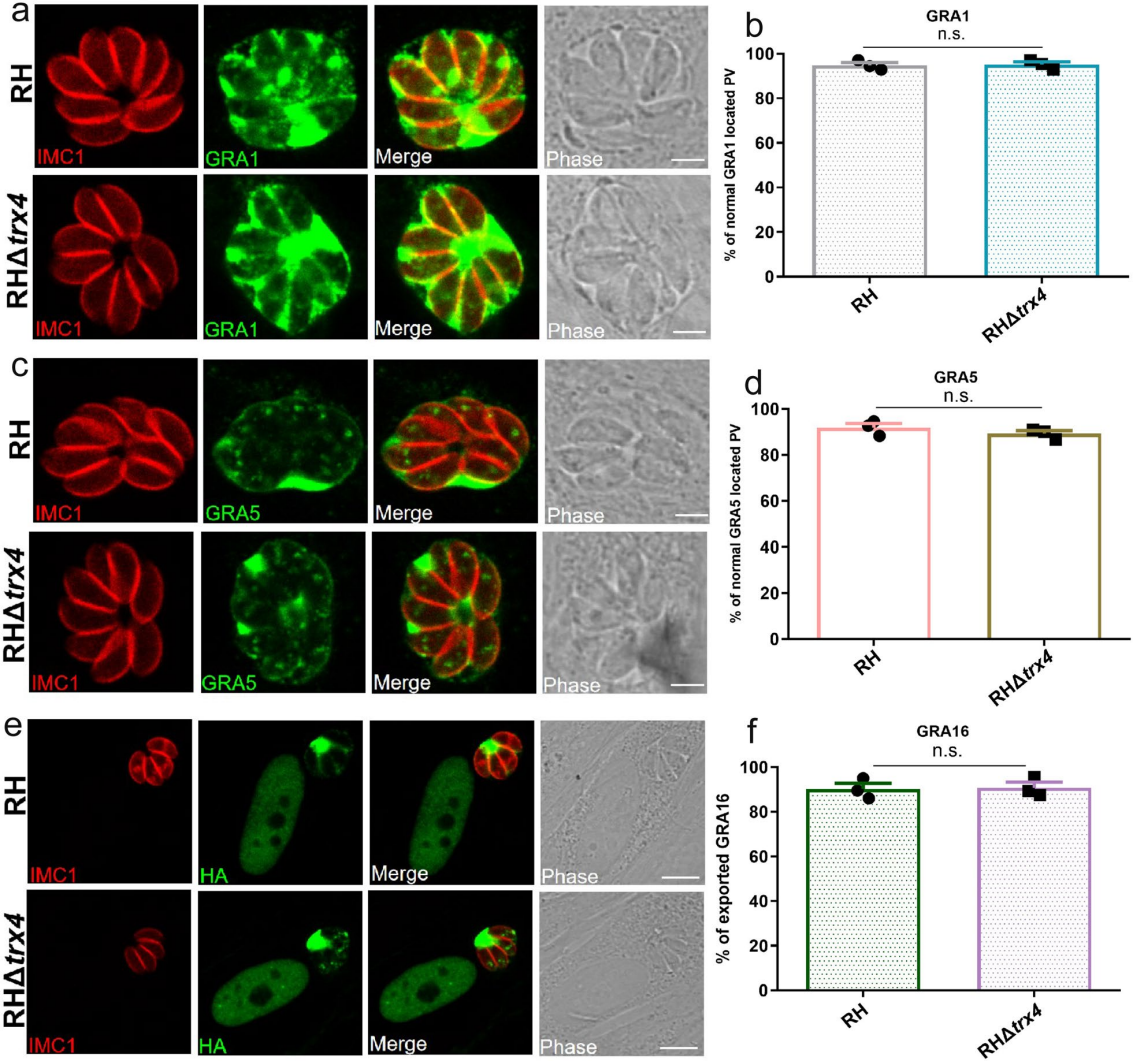
The expression patterns of selected GRAs in both RH and *trx4*-mutant strains examined by using IFA. Quantification of the indicated parasite strains revealed that *trx4* deficiency did not affect the location of GRA1 (**a** and** b**) and GRA5 (**c** and** d**) and did not alter the translocation of GRA16 into host cell nucleus (**e** and** f**). Red, mouse anti-IMC1 or rabbit anti-IMC1; green, mouse anti-GRA1, rabbit anti-GRA5 or rabbit anti-HA. Scale bar: 3 μm. The experiments were performed three independent times. The difference was analyzed by Student’s *t*-test. n.s., not significant

Several PV-located proteins that serve as chaperones or participate in protein translocation such as GRA44 and MYR1 [24] were found to be Trx4 proximity proteins. To assess whether the deletion of *trx4* affected the translocation of GRA proteins, *trx4*-deficient parasites were subjected to staining with mouse anti-GRA1 or rabbit anti-GRA5. IFA results showed that the expression of GRA1 (Fig. 6a and b) and GRA5 (Fig. 6c and d) remained unaffected. Moreover, plasmids expressing GRA16-HA were transiently transfected into both RH and RHΔ*trx4* strains to assess whether the deletion of *trx4* affects the export of GRA16. The results demonstrated that the export and localization of GRA16 were similar between the RH and RHΔ*trx4* strains (Fig. 6e and f).

### *Trx4* disruption attenuated the parasite virulence

**Fig. 7:**
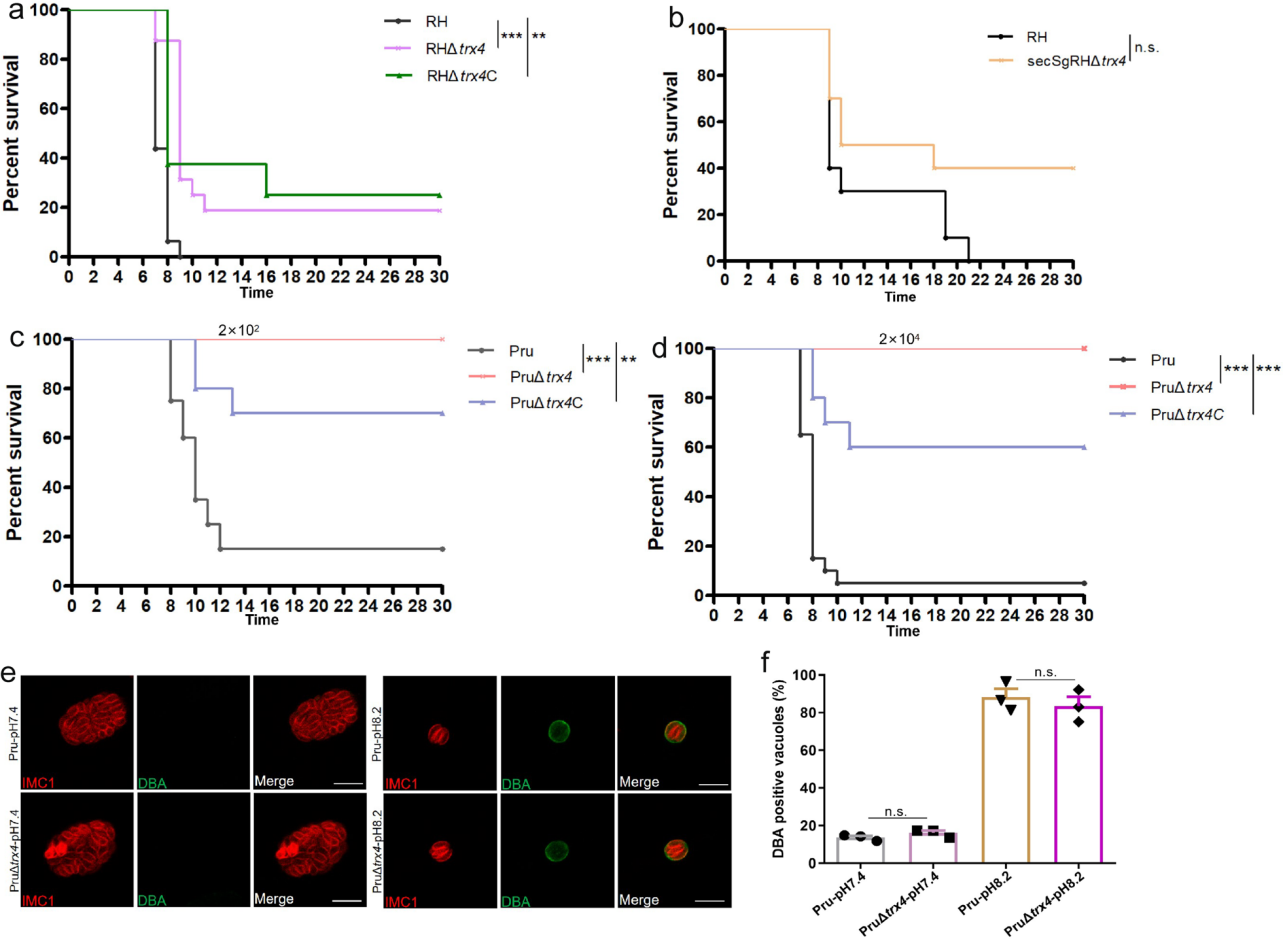
Virulence assays of *trx4-*deficient parasites in RH strain and Pru strain. **a** Survival of Kunming mice that were infected by 2 × 10^2^ dose of RH (*n* = 16 mice), RHΔ*trx4* (*n* = 16 mice) or RHΔ*trx4*C (*n* = 8 mice). Survival curves of mice were analyzed by Gehan-Breslow-Wilcoxon test. **P < 0.01; ***P < 0.001. **b** Survival of Kunming mice that were infected by 2 × 10^2^ dose of RH (*n* = 10 mice) or secSgRHΔ*trx4* (*n* = 10 mice). Survival curves of mice were analyzed by Gehan-Breslow-Wilcoxon test. ns., not significant **c** and **d** Survival of Kunming mice that were infected by low dose (2 × 10^2^) of Pru strains (*n* = 20 mice/Pru strain; *n* = 20 mice/PruΔ*trx4* strain; *n* = 10 mice/PruΔ*trx4*C strain) (**c**) or high dose (2 × 10^4^) of Pru strains (*n* = 20 mice/Pru strain; *n* = 20 mice/PruΔ*trx4* strain; *n* = 10 mice/PruΔ*trx4*C strain) (**d**). Survival curves of mice were analyzed by Gehan-Breslow-Wilcoxon test. **P < 0.01; ***P < 0.001. **e** Representative IFA images of bradyzoite differentiation conducted in vitro using the Pru and PruΔ*trx4* strains. Green, fluorescein-*Dolichos biflorus* agglutinin (DBA); red, rabbit anti-IMC1. Scale bar: 10 μm. (**e**) Differences in the proportion of bradyzoite differentiation between Pru and PruΔ*trx4* strains when parasites were cultured at pH7.4 or pH8.2 for 2 days. The experiments were performed three independent times. The differences were analyzed by Student’s *t* test. n.s., not significant

To determine whether Trx4 plays a role in the parasite pathogenicity, Kunming mice were infected by 2 × 10^2^ tachyzoites of RH, RHΔ*trx4* or RHΔ*trx4*C and monitored for survival. Results showed that deletion of *trx4* attenuated the virulence of RH strain; however, RHΔ*trx4*C did not completely restore this defect (Fig. 7a). Additionally, the virulence of secSgRHΔ*trx4*, which was deleted by a second SgRNA, was also evaluated. The results showed that mice infected with RH strain reached the humane endpoint within 21 days of infection, while 40% of the mice infected with secSgRHΔ*trx4* were still alive until 30 days of infection, suggesting that deletion of *trx4* by the second SgRNA could attenuate the RH virulence, although the survival curves between the two groups were not significantly different, which might due to individual differences between mice (Fig. 7b).

Regarding the type II strain, Kunming mice were infected by a low dose (2 × 10^2^) or high dose (2 × 10^4^) of tachyzoites of the Pru strain and monitored for survival. Regardless of the infection dose, the survival rate between the Pru and PruΔ*trx4* strains was significantly different, with all mice infected by PruΔ*trx4* remaining alive until 30 days post infection (Fig. 7c and d). Moreover, PruΔ*trx4*C partly restored the virulence (Fig. 7c and d). Brain cyst burden was assessed in the mice that remained alive at day 30 post infection, and the results indicated that fewer brain cysts were observed in mice infected by either a low dose (6.7 ± 2.5) or high dose (7.1 ± 3.1) of the KO strain, whereas mice infected by either the low or high dose of wt parasites had 77.67 ± 14.68 brain cysts or 200 brain cysts, respectively. Moreover, PruΔ*trx4*C restored the defect of cyst formation in vivo, with mice infected by either the low or high dose of PruΔ*trx4*C having 114.3 ± 20.72 cysts or 163.8 ± 26.07 cysts, respectively.

To assess whether the observed defect in cyst formation in vivo for PruΔ*trx4* was a result of the parasites’ inability to form cysts, we evaluated the rate of PruΔ*trx4* transformation into cysts in vitro (Fig. 7e). Results showed that the rates of DBA-positive vacuoles were not significantly different between wt and KO strains when the parasites were cultured at 37 ℃ in a 5% CO_2_ atmosphere or induced by differentiation medium (pH = 8.2, ambient CO_2_) for 2 days (Fig. 7f).

## Discussion

In this work, we have identified the Trx4 protein of *T. gondii* as a novel thioredoxin protein of 622 amino acids with two Trx domains. The newly described protein TgTrx4 was expressed in the PV and co-localized with GRA1 and GRA5. In *P. falciparum*, thioredoxin 2 (PfTrx2) is a member of a protein complex embedded in the PVM that plays a role in the translocation of secretory proteins [28]. Disruption of PfTrx2 affects the parasite's ability to secrete proteins into the host cells [31]. The localization of Trx2 in *P. falciparum* is similar to that of Trx4 in *T. gondii*. However, the two proteins have different homologies. PfTrx2 has a molecular weight of 19 kDa, while TgTrx4 has a molecular weight of 69 kDa, suggesting that these proteins may exert different functions in the respective parasite species.

Consistent with its negative phenotypic value (− 1.29), the deletion of *trx4* impaired the parasite invasion into the host cells as shown by the smaller plaques produced by the KO strain compared with that produced by the wt strain in both RH and Pru strains. To further characterize the phenotypic changes caused by *trx4* deficiency, we constructed the complemented strains in RH and Pru strains, namely RHΔ*trx4*C-6HA and PruΔ*trx4*C-6HA, respectively, in which the C-terminus of Trx4 was fused with a 6 × HA tag and a DHFR terminator. However, these complemented strains only restored the expression of Trx4 and did not restore their biological function. It is unclear, though, why the two complemented strains failed to restore the protein function. It is possible that this is caused by modification introduced by the HA tag inserted at the C-terminus of Trx4 or the heterologous DHFR terminator. The complemented strains, RHΔ*trx4*C and PruΔ*trx4*C, with wt Trx4 were also constructed, where Trx4 was expressed by its own promoter and terminated by its own terminator. Fortunately, both RHΔ*trx4*C and PruΔ*trx4*C restored the defects of cell invasion and plaque formation, suggesting that impairment in the Trx4 function can be attributed to the HA tag inserted at the C-terminus of Trx4 or the heterologous terminator. Notably, the newly constructed strain using different guide RNAs (gRNA) exhibited growth defects similar to those observed in the original lines, suggesting that the observed growth defects were primarily attributed to the loss of *trx4* and were not the result of off-target effects. Mutation of Trx domains in Trx4 in RHΔ*trx4*C strains were constructed. Phenotypic analysis revealed that both Trx domains were important for the invasion and plaque formation of RH strain, suggesting Trx domains played roles in the biological function of Trx4. On the other hand, insertion of the 6 × HA tag may affect the localization of Trx4, as observed by the inability of complemented strains RHΔ*trx4*C-6HA and PruΔ*trx4*C-6HA to restore the function of Trx4. This suggests that modification of the C-terminus is apparently not tolerated, warranting further study on the localization of Trx4 using anti-Trx4 antibody.

Traditional co-immunoprecipitation analysis, commonly used to characterize biological functions, is limited by transient or weak interactions [36]. Therefore, enzyme-catalytic proximity labeling techniques, coupled with mass spectrometry, have emerged as efficient tools for dissecting protein-protein interactions and organelle composition. The two most notable systems are BioID and TurboID [34]. Given the specific localization of Trx4, we employed the TurboID labeling technique to identify candidate proximity proteins of Trx4. Trx4, tagged with TurboID, was expressed in the PV, which was similar to Trx4 tagged with HA tag. The majority of proximity proteins were biotinylated by D-biotin and were co-localized with Trx4. Following mass spectrometry analysis, a total of 69 proteins were identified as interesting candidates, with the vast majority (39 out of 69) being located in dense granules. As anticipated, many of these dense granule-localized proteins were previously reported GRAs, including GRA16, GRA17, GRA39, GRA57, GRA70 and others [41–43].

Interestingly, Trx4 was also found as proximity protein of GRA17, which is localized at dense granules, further confirming Trx4 as a dense granule protein [26]. Biotinylation of proximity proteins using Trx4-TurboID* also revealed the presence of MYR1, MYR3 and MYR4, which are important for the translocation of GRAs [44, 45]. Our results also identified PPM3C, which interacts with MYR1. PPM3C plays an essential role in GRA16 translocation [25]. However, deletion of *trx4* did not have any effect on the translocation of GRA16, suggesting that Trx4 was not involved in the translocation of GRAs. To further validate the mass spectrometry results, we focused on three novel proteins (TGGT1_215910, TGGT1_216720 and TGGT1_225160) identified in the data sets of the Trx4-TurboID* strain, which had not been previously characterized. We explored their localization within the Trx4-6HA strain. These hypothetical proteins were co-localized with Trx4 and were also expressed in the PV. We individually inserted a 2 × Ty tag at the C-terminus of TGGT1_215910, TGGT1_216720 or TGGT1_225160 in both wt and RHΔ*trx4* backgrounds. However, the distribution of these PV-resident proteins remained unaffected regardless of whether Trx4 was present.

Deletion of *trx4* impaired the tachyzoite ability to invade host cells in both RH (Type I) and Pru (Type II) strains in vitro, which may contribute to the attenuated virulence in mice. *Toxoplasma gondii* RH strain of the virulent type I displays enhanced migratory capacity, kills mice in the acute stage and does not readily form tissue cyst in vivo. The type II Pru strain is less virulent and forms cysts in the brain, skeletal muscle and other tissues of the host, leading to a latent infection. The disruption of *trx4* significantly attenuated parasite virulence and reduced the number of brain cysts, suggesting that Trx4 may play a role in the pathogenicity and persistence of *T. gondii* within the host. Complementation of Trx4 at the *uprt* locus did not completely restore virulence, suggesting that the endogenous site of Trx4 may affect its activity in vivo. Reassuringly, while this manuscript was under revision, Tachibana et al. (bioRxiv preprint) identified TGGT1_224060 as a virulence factor by CRISPR screening, showing that deletion of TGGT1_224060 attenuated the virulence of RH strain [46].

The PV is a critical site for the complex interactions that occur between *T. gondii* and the host cell and plays a central role in modulating nutrient acquisition and resistance to the host cell's IRGs defense system. The fact that Trx4 is predominantly expressed in the PV and associates with many proteins localized in this compartment indicates that Trx4 may play a role in regulating pathways related to the parasite ability to interact with and modulate cell processes specific to the PV. However, this merits further investigations.

## Conclusions

In this study, a newly discovered thioredoxin (Trx4) of *T. gondii* was characterized. Trx4 was localized in the PV. The deletion of *trx4* significantly impaired the parasite's ability to invade the host cells. Using the TurboID system, we identified many proximity proteins associated with Trx4, and a significant proportion of these proteins was located in the PV. The deletion of *trx4* significantly reduced the parasite virulence and decreased cyst formation in the brain of mice. Our data provided insight into the role of Trx4 in *T. gondii* and highlighted its significance in the parasite invasion and virulence. The association of Trx4 with many proximity proteins localized in the PV clearly implicates this protein in multiple processes relevant to this unique vacuolar compartment. Further studies are needed to elucidate the mechanisms underlying these findings and the relationships between Trx4 and its associated proteins.

### Supplementary Information

**Additional file 1: Table S1.** Primers used in this study.

**Additional file 2: Table S2.** Information about *Toxoplasma gondii* thioredoxins available in ToxoDB.

**Additional file 3: Figure S1.** Construction of the C-terminal strains. (a) Schematic diagram shows the construction of RHTrx4-6HA strain. (b–d) IFA shows that Trx4 was not localized in the apicoplasts (b), mitochondria (c) and micronemes (d), with these organelles being stained with ACP, hsp60 and MIC2, respectively. Green, rabbit anti-ACP, rabbit anti-hsp60 or rabbit anti-MIC2; red, mouse anti-HA. Scale bar: 2 μm.

**Additional file 4: Figure S2.** Construction of the complemented strains in RH strain. (a) Schematic diagram shows the construction of *trx4*-deficient strain. (b) IFA revealed that RHΔ*trx4*C-6HA restored the expression of Trx4. Red, mouse anti-IMC1; green, rabbit anti-HA. Scale bar: 3 μm. (c) Plaque assays of RH, RHΔ*trx4* and RHΔ*trx4*C-6HA. (d) Schematic diagram of the construction of complemented strain without HA tag, which was named as RHΔ*trx4*C. (e and f) PCR analysis confirmed the construction of RHΔ*trx4* and RHΔ*trx4*C at DNA level (e) and at cDNA level (f). (g and h) PCR analysis confirmed the construction of RHΔ*trx4* that was disrupted by the second SgRNA at DNA level (g) and cDNA level (h). (i) The plaque assay of RH and secSgRHΔ*trx4* that was deleted by the second SgRNA. (j) The relative plaque sizes of RH and secSgRHΔ*trx4* that were measured from (i). The experiments were performed three independent times. The difference was analyzed by Student’s *t*-test. ***P < 0.001; n.s., not significant. (k) Quantification of invasion ability of RH and secSgRHΔ*trx4* strains. The experiments were performed three independent times. Five microscopic fields were randomly selected in each sample. The difference was analyzed by Student’s *t*-test. *P < 0.05; n.s., not significant. (l) Quantification of replication efficiency of RH and secSgRHΔ*trx4* strains grown in HFFs for 24 h. The experiments were performed three independent times. The difference was analyzed by Student’s *t*-test. n.s., not significant. (m) Quantification of the egress ability of RH and secSgRHΔ*trx4* strains. The experiments were performed three independent times. The difference was analyzed by Student’s *t*-test. n.s., not significant.

**Additional file 5: Figure S3.** Phenotypic analysis of PruΔ*trx4*. (a and b) PCR analysis confirmed the construction of PruΔ*trx4* and the complemented strain that lacks HA tag was named PruΔ*trx4*C at DNA level (a) and cDNA level (b). (c) The plaque assays of Pru, PruΔ*trx4* and PruΔ*trx4*C. White arrow: plaques produced by PruΔ*trx4.* (d) Quantification of the relative plaque sizes produced by Pru, PruΔ*trx4* and PruΔ*trx4*C. The experiments were performed three independent times. The difference was analyzed by Student’s *t*-test. ***P < 0.001; n.s., not significant. (e) Differences in the host cell invasion between Pru, PruΔ*trx4* and PruΔ*trx4*C. Five microscopic fields were randomly selected in each sample. The experiments were performed three independent times. The difference was analyzed by Student’s *t*-test. *P < 0.05; n.s., not significant. (f) Differences in the intracellular replication between Pru, PruΔ*trx4* and PruΔ*trx4*C. The experiments were performed three independent times. The difference was analyzed by Student’s *t*-test. n.s., not significant. (g) Differences in the egress between Pru, PruΔ*trx4* and PruΔ*trx4*C. The experiments were performed three independent times. The difference was analyzed by Student’s *t*-test. n.s., not significant. (h) Plaque assays of Pru, PruΔ*trx4* and PruΔ*trx4*C-6HA. (i) IFA revealed that PruΔ*trx4*C-6HA restored the expression of Trx4. Red, mouse anti-IMC1; green, rabbit anti-HA. Scale bar: 3 μm.

**Additional file 6: Figure S4.** Construction of mutation in the Trx domains in Trx4 in RH strain. (**a**) Schematic diagram shows the construction of Trx-domain mutants in RHΔ*trx4*C strain. (**b**) PCR analysis confirmed that Trx domain was deleted in RHΔ*trx4*C strain. RHΔ*trx4*C-I-dele, RHΔ*trx4*C-II-dele and RHΔ*trx4*C-I + II-dele denote deletion of the first, the second and both Trx domains, respectively.

**Additional file 7: Table S3.** The proximity proteins of Trx4 identified by mass spectrometry.

## Data Availability

The datasets supporting the findings of this article are included within the paper and its Additional materials.

## Abbreviations

EDTA: Ethylenediamine tetra-acetic acid
FBS: Fetal bovine serum
GRA: Dense granule protein
HFF: Human foreskin fibroblast
IFA: Indirect immunofluorescence analysis
KO: Knock out
PTEX: *Plasmodium* translocon of exported proteins
PV: Parasitophorous vacuole
SDS-PAGE: Sodium dodecyl sulfate polyacrylamide gel electrophoresis
wt: Wild type

## References

[1] Robert-Gangneux F, Dardé ML (2012). Epidemiology of and diagnostic strategies for toxoplasmosis. Clin Microbiol Rev.

[2] Elsheikha HM, Marra CM, Zhu XQ (2021). Epidemiology, pathophysiology, diagnosis, and management of cerebral toxoplasmosis. Clin Microbiol Rev.

[3] Milne G, Webster JP, Walker M (2020). *Toxoplasma gondii*: an underestimated threat?. Trends Parasitol.

[4] Matta SK, Rinkenberger N, Dunay IR, Sibley LD (2021). *Toxoplasma gondii* infection and its implications within the central nervous system. Nat Rev Microbiol.

[5] Smith NC, Goulart C, Hayward JA, Kupz A, Miller CM, van Dooren GG (2021). Control of human toxoplasmosis. Int J Parasitol.

[6] Cabral CM, Tuladhar S, Dietrich HK, Nguyen E, MacDonald WR, Trivedi T (2016). Neurons are the primary target cell for the brain-tropic intracellular parasite *Toxoplasma gondii*. PLoS Pathog.

[7] Zhao XY, Ewald SE (2020). The molecular biology and immune control of chronic *Toxoplasma gondii* infection. J Clin Invest.

[8] Zhang Y, Lai BS, Juhas M, Zhang Y (2019). *Toxoplasma gondii* secretory proteins and their role in invasion and pathogenesis. Microbiol Res.

[9] Clough B, Frickel EM (2017). The *Toxoplasma* parasitophorous vacuole: an evolving host-parasite frontier. Trends Parasitol.

[10] Hakimi MA, Olias P, Sibley LD (2017). *Toxoplasma* effectors targeting host signaling and transcription. Clin Microbiol Rev.

[11] Lima TS, Lodoen MB (2019). Mechanisms of human innate immune evasion by *Toxoplasma gondii*. Front Cell Infect Microbiol.

[12] Wang Y, Sangaré LO, Paredes-Santos TC, Saeij JPJ (2020). *Toxoplasma* mechanisms for delivery of proteins and uptake of nutrients across the host-pathogen interface. Annu Rev Microbiol.

[13] Deffieu MS, Alayi TD, Slomianny C, Tomavo S (2019). The *Toxoplasma gondii* dense granule protein TgGRA3 interacts with host Golgi and dysregulates anterograde transport. Biol Open.

[14] Rezaei F, Sharif M, Sarvi S, Hejazi SH, Aghayan S, Pagheh AS (2019). A systematic review on the role of GRA proteins of *Toxoplasma gondii* in host immunization. J Microbiol Methods.

[15] Fentress SJ, Behnke MS, Dunay IR, Mashayekhi M, Rommereim LM, Fox BA (2010). Phosphorylation of immunity-related GTPases by a *Toxoplasma gondii*-secreted kinase promotes macrophage survival and virulence. Cell Host Microbe.

[16] Steinfeldt T, Könen-Waisman S, Tong L, Pawlowski N, Lamkemeyer T, Sibley LD (2010). Phosphorylation of mouse immunity-related GTPase (IRG) resistance proteins is an evasion strategy for virulent *Toxoplasma gondii*. PLoS Biol.

[17] Alaganan A, Fentress SJ, Tang K, Wang Q, Sibley LD (2014). *Toxoplasma* GRA7 effector increases turnover of immunity-related GTPases and contributes to acute virulence in the mouse. Proc Natl Acad Sci U S A.

[18] Hermanns T, Müller UB, Könen-Waisman S, Howard JC, Steinfeldt T (2016). The *Toxoplasma gondii* rhoptry protein ROP18 is an Irga6-specific kinase and regulated by the dense granule protein GRA7. Cell Microbiol.

[19] Fleckenstein MC, Reese ML, Könen-Waisman S, Boothroyd JC, Howard JC, Steinfeldt T (2012). A *Toxoplasma gondii* pseudokinase inhibits host IRG resistance proteins. PLoS Biol.

[20] Niedelman W, Gold DA, Rosowski EE, Sprokholt JK, Lim D, Farid Arenas A (2012). The rhoptry proteins ROP18 and ROP5 mediate *Toxoplasma gondii* evasion of the murine, but not the human, interferon-gamma response. PLoS Pathog.

[21] Etheridge RD, Alaganan A, Tang K, Lou HJ, Turk BE, Sibley LD (2014). The *Toxoplasma* pseudokinase ROP5 forms complexes with ROP18 and ROP17 kinases that synergize to control acute virulence in mice. Cell Host Microbe.

[22] Singh S, Murillo-León M, Endres NS, Arenas Soto AF, Gómez-Marín JE, Melbert F (2023). ROP39 is an Irgb10-specific parasite effector that modulates acute *Toxoplasma gondii* virulence. PLoS Pathog.

[23] Nyonda MA, Hammoudi PM, Ye S, Maire J, Marq JB, Yamamoto M (2021). *Toxoplasma gondii* GRA60 is an effector protein that modulates host cell autonomous immunity and contributes to virulence. Cell Microbiol.

[24] Cygan AM, Theisen TC, Mendoza AG, Marino ND, Panas MW, Boothroyd JC (2020). Coimmunoprecipitation with MYR1 identifies three additional proteins within the *Toxoplasma gondii* parasitophorous vacuole required for translocation of dense granule effectors into host cells. mSphere.

[25] Mayoral J, Tomita T, Tu V, Aguilan JT, Sidoli S, Weiss LM (2020). *Toxoplasma gondii* PPM3C, a secreted protein phosphatase, affects parasitophorous vacuole effector export. PLoS Pathog.

[26] Nadipuram SM, Kim EW, Vashisht AA, Lin AH, Bell HN, Coppens I (2016). In vivo biotinylation of the *Toxoplasma* parasitophorous vacuole reveals novel dense granule proteins important for parasite growth and pathogenesis. MBio.

[27] Elsworth B, Matthews K, Nie CQ, Kalanon M, Charnaud SC, Sanders PR (2014). PTEX is an essential nexus for protein export in malaria parasites. Nature.

[28] de Koning-Ward TF, Gilson PR, Boddey JA, Rug M, Smith BJ, Papenfuss AT (2009). A newly discovered protein export machine in malaria parasites. Nature.

[29] Arnér ES, Holmgren A (2000). Physiological functions of thioredoxin and thioredoxin reductase. Eur J Biochem.

[30] Vazquez DS, Delfino JM, Santos J (2015). Thioredoxin from *Escherichia coli* as a role model of molecular recognition, folding, dynamics and function. Protein Pept Lett.

[31] Sharma A, Sharma A, Dixit S, Sharma A (2011). Structural insights into thioredoxin-2: a component of malaria parasite protein secretion machinery. Sci Rep.

[32] Biddau M, Bouchut A, Major J, Saveria T, Tottey J, Oka O (2018). Two essential Thioredoxins mediate apicoplast biogenesis, protein import, and gene expression in *Toxoplasma gondii*. PLoS Pathog.

[33] Zhang ZW, Li TT, Wang JL, Liang QL, Zhang HS, Sun LX (2021). Functional characterization of two thioredoxin proteins of *Toxoplasma gondii* using the CRISPR-Cas9 system. Front Vet Sci.

[34] Xu Y, Fan X, Hu Y (2021). In vivo interactome profiling by enzyme-catalyzed proximity labeling. Cell Biosci.

[35] Kim DI, Birendra KC, Zhu W, Motamedchaboki K, Doye V, Roux KJ (2014). Probing nuclear pore complex architecture with proximity-dependent biotinylation. Proc Natl Acad Sci U S A.

[36] Snider J, Kotlyar M, Saraon P, Yao Z, Jurisica I, Stagljar I (2015). Fundamentals of protein interaction network mapping. Mol Syst Biol.

[37] Branon TC, Bosch JA, Sanchez AD, Udeshi ND, Svinkina T, Carr SA (2018). Efficient proximity labeling in living cells and organisms with TurboID. Nat Biotechnol.

[38] Li S, Liu J, Zhang H, Sun Z, Ying Z, Wu Y (2021). *Toxoplasma gondii* glutathione S-transferase 2 plays an important role in partial secretory protein transport. FASEB J.

[39] Wang JL, Li TT, Elsheikha HM, Liang QL, Zhang ZW, Wang M (2022). The protein phosphatase 2A holoenzyme is a key regulator of starch metabolism and bradyzoite differentiation in *Toxoplasma gondii*. Nat Commun.

[40] Chern JH, Pasquarelli RR, Moon AS, Chen AL, Sha J, Wohlschlegel JA (2021). A novel *Toxoplasma* inner membrane complex suture-associated protein regulates suture protein targeting and colocalizes with membrane trafficking machinery. MBio.

[41] Seo SH, Kim SG, Shin JH, Ham DW, Shin EH (2020). *Toxoplasma* GRA16 inhibits NF-κB activation through PP2A-B55 upregulation in non-small-cell lung carcinoma cells. Int J Mol Sci.

[42] Gold DA, Kaplan AD, Lis A, Bett GC, Rosowski EE, Cirelli KM (2015). The *Toxoplasma* dense granule proteins GRA17 and GRA23 mediate the movement of small molecules between the host and the parasitophorous vacuole. Cell Host Microbe.

[43] Lockyer EJ, Torelli F, Butterworth S, Song OR, Howell S, Weston A (2023). A heterotrimeric complex of *Toxoplasma* proteins promotes parasite survival in interferon gamma-stimulated human cells. PLoS Biol.

[44] Marino ND, Panas MW, Franco M, Theisen TC, Naor A, Rastogi S (2018). Identification of a novel protein complex essential for effector translocation across the parasitophorous vacuole membrane of *Toxoplasma gondii*. PLoS Pathog.

[45] Franco M, Panas MW, Marino ND, Lee MC, Buchholz KR, Kelly FD (2016). A novel secreted protein, MYR1, is central to *Toxoplasma*’s manipulation of host cells. MBio.

[46] Tachibana Y, Sasai M, Yamamoto M. In vivo CRISPR screens identify novel virulence genes among proteins of unassigned subcellular localization in *Toxoplasma*. 2024. 10.1101/2024.01.28.577556. 10.1128/mbio.01728-24 PMC11389413 39082802

