# Supplementary material for: Trx4, a novel thioredoxin protein, is important for Toxoplasma gondii fitness
Source: Parasit Vectors. 2024 Apr 4;17:178. doi: 10.1186/s13071-024-06259-9 (PMC10996207; doi:10.1186/s13071-024-06259-9)
Supplement: Supplementary file 1 — Additional file 1: Table S1. Primers used in this study. [file 13071_2024_6259_MOESM1_ESM.docx]

**Additional file 1: Table S1 Primers used in this study**

| Primer name | Primer sequence (5′-3′) |
| --- | --- |
| Trx4-SgRNA1 | GTGATGTCCAATCGCGACCA |
| Trx4-SgRNA2 | GCGTCGAAGATGGAGACGAG |
| Trx4-KO-F | GAGCACATTCGCCGCCTTCTG |
| Trx4-KO-R | GAAACTTGAGCGCAGGGTGGTGT |
| U5Trx4-Gibson-F | GGTTTTCCCAGTCACGACGTTGAAACCCGCCGTTAAGTCTTCTCA |
| U5Trx4-Gibson-R | GGATTTACAGCCTGGCGAAGCTTCGTCGCCTTGGACCAGTAGCA |
| U3Trx4-Gibson-F | CTATGCACTTGCAGGATGAATTCCGGTGAAGACTGTCGTGGGATC |
| U3Trx4-Gibson-R | GAGCGGATAACAATTTCACAGGCGTTCAAACCTGGACTGGA |
| Trx4-3-SgRNA | AGCCGTCGGTAGTGTCCTGG |
| 3′ Trx4-KO-F | GGTATCCCACGATCTTCCTGTTTC |
| 3′ Trx4-KO-R | CCTGTCTTGGTTGCACGTTGTTC |
| Trx4-3-HRF | AACCGCGGCGGACAAGCCGTCGGTAGTGTCCTGGAGGAACTGGCTAGCAAGGGCTCGGG |
| Trx4-3-HRR | CTGTTCTCCCTCGTTCTCTACGTCTGGTCTACAGTTCCTCCAATACGACTCACTATAGG |
| PTrx4-Gibson-F | AGTGGAGGACGGGAATTCGGGCCCGTTAACGTGCATCCAAGCAGTAGT |
| PTrx4-Gibson-R | GTTGCGAAGAAATGAACCGAGCGT |
| Trx4-CDS-Gibson-F | ACGCTCGGTTCATTTCTTCGCAACATGGCGCCTCGCACCTCTCTCGCG |
| Trx4-CDS-Gibson-R | GGTCGAGCCCGAGCCCTTGCTAGCAAGTTCCTCCAGGACACTACCGAC |
| Promoter-KZ-Trx4-F | GTTAACGTGCATCCAAGCAGTAGT |
| Terminator-KZ-Trx4-R | CGATCTGTTCTCCCTCGTTCTCTACGTCTGGTCTACAGTTCC |
| Trx4-I-Trx-deletion-F | CGCGACAAGTCTCTGACTTC |
| Trx4-I-Trx-deletion-R | GCTGTAGCTCTTCGTCGACT |
| Trx4-II-Trx-deletion-F | CGCGCCCACTTCGACACAGGCAA |
| Trx4-II-Trx-deletion-R | CTCGGAGCGGTAGTACGGCT |
| TGGT1_215910-3-SgRNA | ACTGCTTCTGAAAAGCTTGT |
| 3′ TGGT1_215910-KO-F | CGTTAATAGGGCGTTTGTC |
| 3′ TGGT1_215910-KO-R | AGGGTTTACCGCTTCGTT |
| TGGT1_216720-3-SgRNA | GTCAACGACGGCTGACAGCT |
| 3′ TGGT1_216720-KO-F | GTATGAAAACGACTTGAATG |
| 3′ TGGT1_216720-KO-R | GTCTGTCTCGACCTGGAA |
| TGGT1_225160-3-SgRNA | ACGTTGAAGCCTGTTGTTTG |
| 3′ TGGT1_225160-KO-F | CCGAGAAGTTGGAAGACG |
| 3′ TGGT1_225160-KO-R | ACGAGACGAGGAAGGTTT |
