# Supplementary material for: Trx4, a novel thioredoxin protein, is important for Toxoplasma gondii fitness
Source: Parasit Vectors. 2024 Apr 4;17:178. doi: 10.1186/s13071-024-06259-9 (PMC10996207; doi:10.1186/s13071-024-06259-9)
Supplement: Supplementary file 2 — Additional file 2: Table S2. Information about Toxoplasma gondii thioredoxins available in ToxoDB. [file 13071_2024_6259_MOESM2_ESM.docx]

| **Gene ID** | **Product description^a^** | **Predicted location^b^** | **GWCS^c^** | **Exons** | **Molecular weight (kDa)** |
| --- | --- | --- | --- | --- | --- |
| TGGT1_293870 | **Trx1**, thioredoxin, putative | Cytosol | -4.53 | 3 | 12 |
| TGGT1_291810 | **Trx2**, thioredoxin domain-containing protein | ER | -0.76 | 3 | 25 |
| TGGT1_247350 | **Trx3**, thioredoxin domain-containing protein | ER | 0.8 | 4 | 34 |
| TGGT1_224060 | **Trx4**, thioredoxin, putative | Golgi PM-integral  PM-peripheral 2 | -1.29 | 1 | 69 |
| TGGT1_270120 | **TLP1**, thioredoxin-like protein TLP1 | Mitochondrion | -1.61 | 5 | 27 |
| TGGT1_290260 | **CTrp26**, thioredoxin family Trp26 protein | Cytosol | 0.42 | 8 | 24 |
| TGGT1_266620 | **CTrx1**, thioredoxin domain-containing protein | Cytosol | 1.28 | 4 | 23 |
| TGGT1_209950 | thioredoxin, putative | Apicoplast | -5.2 | 7 | 48 |
| TGGT1_308050 | thioredoxin domain-containing protein | None | -3.5 | 5 | 23 |
| TGGT1_216510 | thioredoxin, putative | Mitochondrion | -4.18 | 9 | 79 |
| TGGT1_247660 | thioredoxin domain-containing protein | None | 1.04 | 5 | 56 |
| TGGT1_255480 | thioredoxin domain-containing protein | None | -0.61 | 4 | 51 |
| TGGT1_204480 | thioredoxin domain-containing protein | Apicoplast | -3.49 | 12 | 72 |
| TGGT1_312110 | ATrx1, apicoplast-associated thioredoxin family protein Atrx1 | Apicoplast | -4.16 | 10 | 87 |
| TGGT1_310770 | ATrx2, apicoplast-associated thioredoxin family protein Atrx2 | Apicoplast | -2.87 | 5 | 39 |

**Additional file 2: Table S2 Information of *Toxoplasma gondii* thioredoxins available in ToxoDB.**

**a,** The bold names of the thioredoxins were designated in this study.

**b,** The protein locations were predicted by hyperLOPIT.

**c,** Phenotype score determined based on a genome-wide CRISPR/Cas9 screening.
