# Supplementary figures and images for: Trx4, a novel thioredoxin protein, is important for Toxoplasma gondii fitness
Source: Parasit Vectors. 2024 Apr 4;17:178. doi: 10.1186/s13071-024-06259-9 (PMC10996207; doi:10.1186/s13071-024-06259-9)

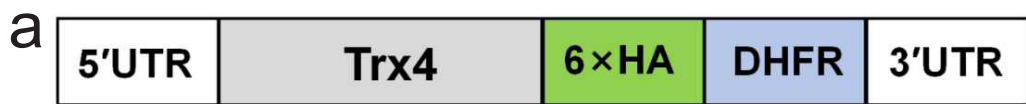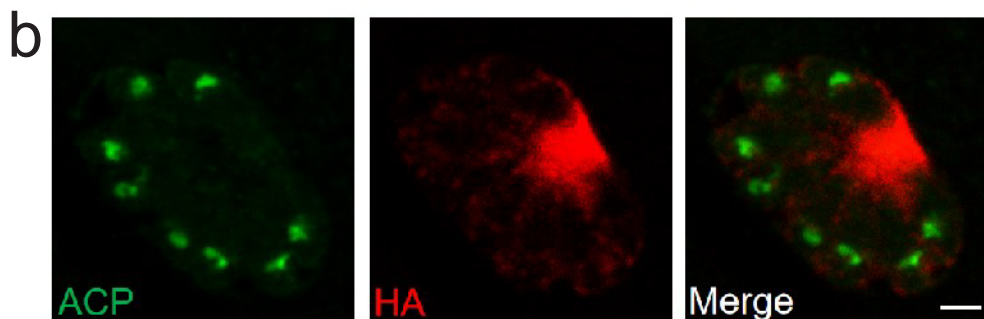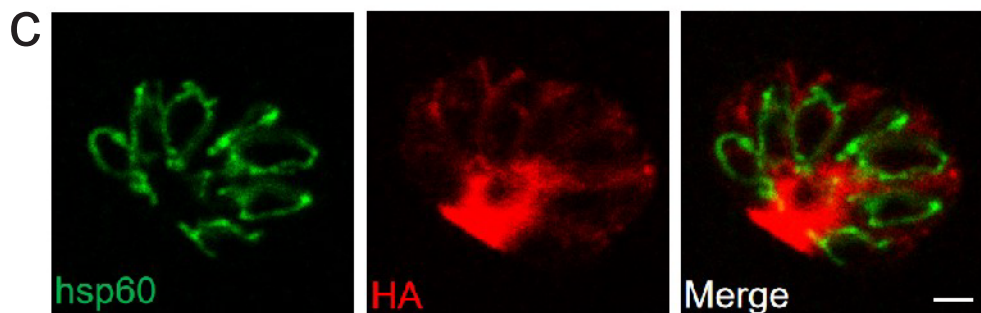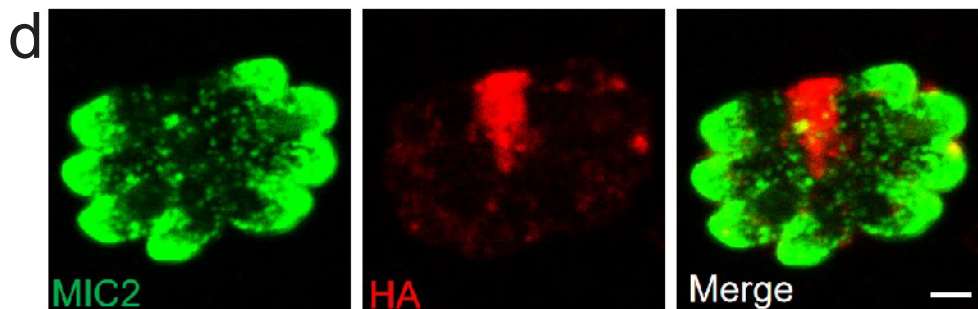

Supplement: Supplementary file 3 — Additional file 3: Figure S1. Construction of the C-terminal strains. (a) Schematic diagram shows the construction of RHTrx4-6HA strain. (b–d) IFA shows that Trx4 was not localized in the apicoplasts (b), mitochondria (c) and micronemes (d), with these organelles being stained with ACP, hsp60 and MIC2, respectively. Green, rabbit anti-ACP, rabbit anti-hsp60 or rabbit anti-MIC2; red, mouse anti-HA. Scale bar: 2 μm. [file 13071_2024_6259_MOESM3_ESM.pdf]

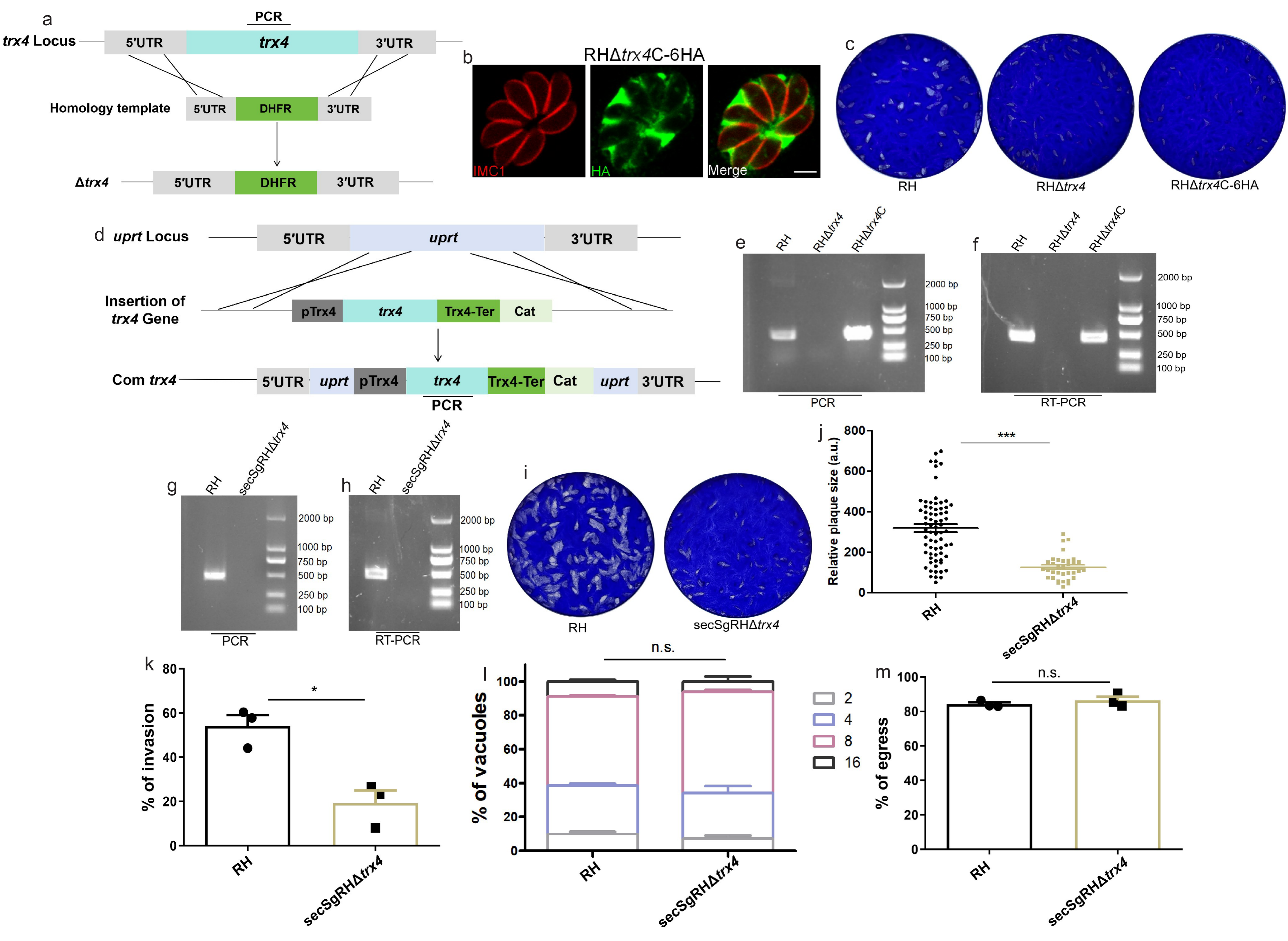

Supplement: Supplementary file 4 — Additional file 4: Figure S2. Construction of the complemented strains in RH strain. (a) Schematic diagram shows the construction of trx4-deficient strain. (b) IFA revealed that RHΔtrx4C-6HA restored the expression of Trx4. Red, mouse anti-IMC1; green, rabbit anti-HA. Scale bar: 3 μm. (c) Plaque assays of RH, RHΔtrx4 and RHΔtrx4C-6HA. (d) Schematic diagram of the construction of complemented strain without HA tag, which was named as RHΔtrx4C. (e and f) PCR analysis confirmed the construction of RHΔtrx4 and RHΔtrx4C at DNA level (e) and at cDNA level (f). (g and h) PCR analysis confirmed the construction of RHΔtrx4 that was disrupted by the second SgRNA at DNA level (g) and cDNA level (h). (i) The plaque assay of RH and secSgRHΔtrx4 that was deleted by the second SgRNA. (j) The relative plaque sizes of RH and secSgRHΔtrx4 that were measured from (i). The experiments were performed three independent times. The difference was analyzed by Student’s t-test. ***P < 0.001; n.s., not significant. (k) Quantification of invasion ability of RH and secSgRHΔtrx4 strains. The experiments were performed three independent times. Five microscopic fields were randomly selected in each sample. The difference was analyzed by Student’s t-test. *P < 0.05; n.s., not significant. (l) Quantification of replication efficiency of RH and secSgRHΔtrx4 strains grown in HFFs for 24 h. The experiments were performed three independent times. The difference was analyzed by Student’s t-test. n.s., not significant. (m) Quantification of the egress ability of RH and secSgRHΔtrx4 strains. The experiments were performed three independent times. The difference was analyzed by Student’s t-test. n.s., not significant. [file 13071_2024_6259_MOESM4_ESM.pdf]

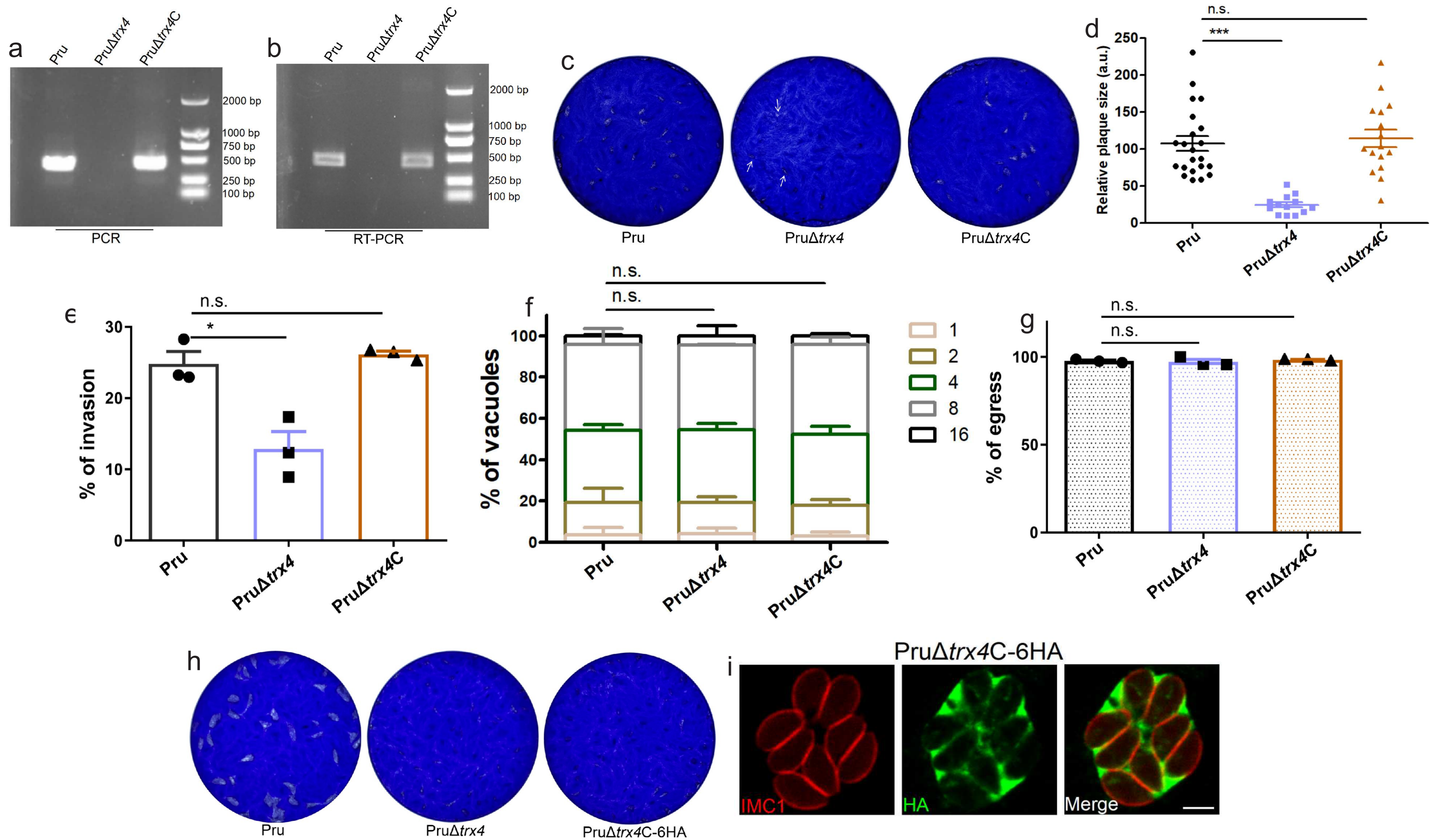

Supplement: Supplementary file 5 — Additional file 5: Figure S3. Phenotypic analysis of PruΔtrx4. (a and b) PCR analysis confirmed the construction of PruΔtrx4 and the complemented strain that lacks HA tag was named PruΔtrx4C at DNA level (a) and cDNA level (b). (c) The plaque assays of Pru, PruΔtrx4 and PruΔtrx4C. White arrow: plaques produced by PruΔtrx4. (d) Quantification of the relative plaque sizes produced by Pru, PruΔtrx4 and PruΔtrx4C. The experiments were performed three independent times. The difference was analyzed by Student’s t-test. ***P < 0.001; n.s., not significant. (e) Differences in the host cell invasion between Pru, PruΔtrx4 and PruΔtrx4C. Five microscopic fields were randomly selected in each sample. The experiments were performed three independent times. The difference was analyzed by Student’s t-test. *P < 0.05; n.s., not significant. (f) Differences in the intracellular replication between Pru, PruΔtrx4 and PruΔtrx4C. The experiments were performed three independent times. The difference was analyzed by Student’s t-test. n.s., not significant. (g) Differences in the egress between Pru, PruΔtrx4 and PruΔtrx4C. The experiments were performed three independent times. The difference was analyzed by Student’s t-test. n.s., not significant. (h) Plaque assays of Pru, PruΔtrx4 and PruΔtrx4C-6HA. (i) IFA revealed that PruΔtrx4C-6HA restored the expression of Trx4. Red, mouse anti-IMC1; green, rabbit anti-HA. Scale bar: 3 μm. [file 13071_2024_6259_MOESM5_ESM.pdf]

**a**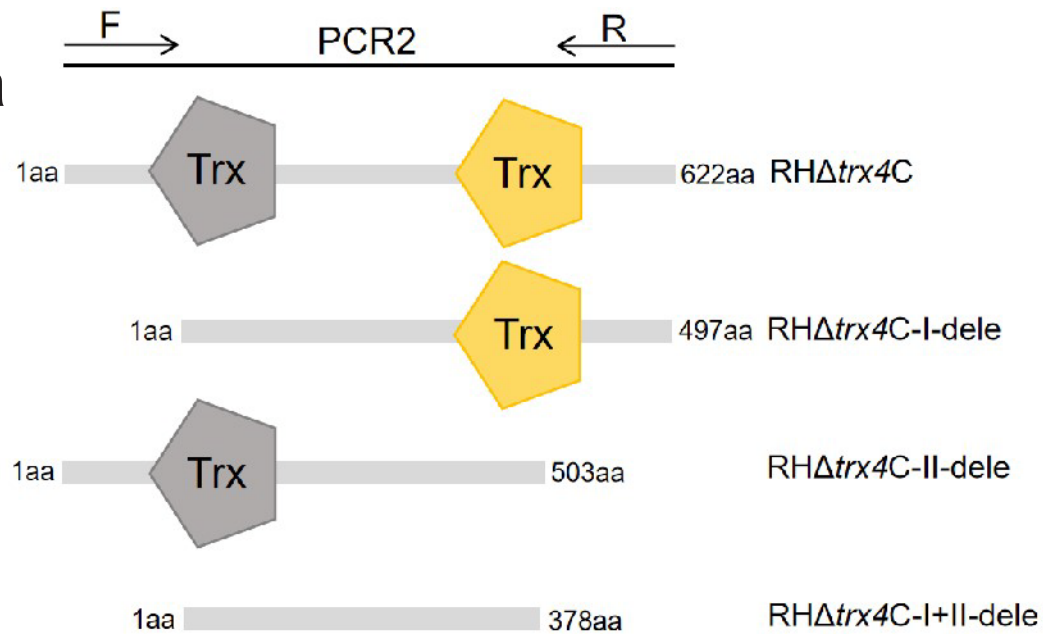**b**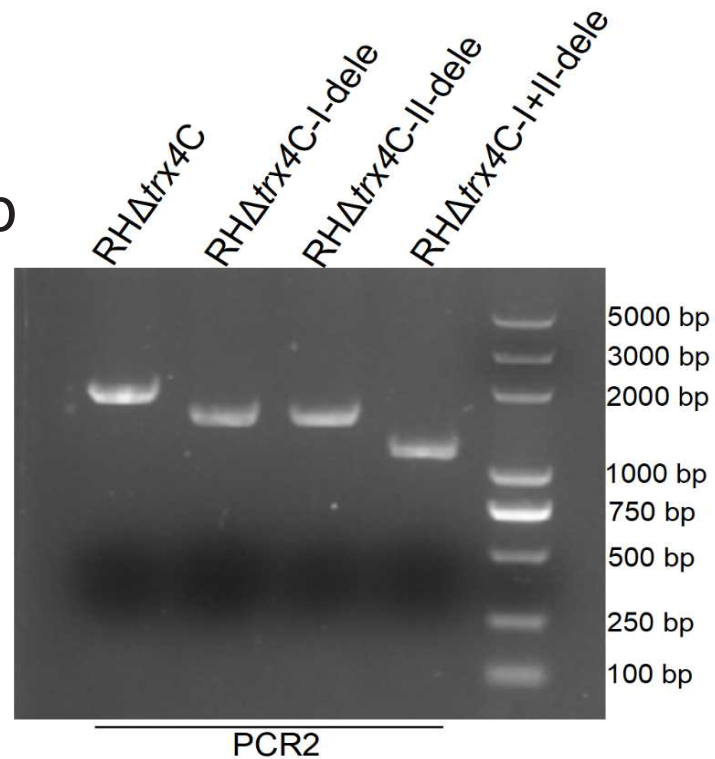

Supplement: Supplementary file 6 — Additional file 6: Figure S4. Construction of mutation in the Trx domains in Trx4 in RH strain. (a) Schematic diagram shows the construction of Trx-domain mutants in RHΔtrx4C strain. (b) PCR analysis confirmed that Trx domain was deleted in RHΔtrx4C strain. RHΔtrx4C-I-dele, RHΔtrx4C-II-dele and RHΔtrx4C-I + II-dele denote deletion of the first, the second and both Trx domains, respectively. [file 13071_2024_6259_MOESM6_ESM.pdf]
